# Supplementary material for: Synergistic dual anion regulation unlocks giant thermopower and power density in hydrogel
Source: Nat Commun. 2026 Mar 30;17:4592. doi: 10.1038/s41467-026-71285-3 (PMC13194681; doi:10.1038/s41467-026-71285-3)
Supplement: Supplementary file 1 — Supplementary Information [file 41467_2026_71285_MOESM1_ESM.pdf]

Supplementary Information

Synergistic dual anion regulation unlocks giant thermopower and power density in hydrogel

Hongbing Li[1], Zhangjie Gu[1], Yaling Zhu[1], Zhaoyang Jiao[1], Jinya Tian[1], Yi Li[1], Yongping Chai[1], Xiaodong Chi[1,2,*]

[1] State Key Laboratory of New Textile Materials and Advanced Processing, School of Materials Science and Engineering, Huazhong University of Science and Technology, Wuhan 430074, China.

[2] Shenzhen Huazhong University of Science and Technology Research Institute, Shenzhen 518000, China

*Corresponding author: xchi@hust.edu.cn.

Supplementary Methods

Experimental methods

Materials. Unless otherwise stated, all chemical reagents and solvents were obtained from commercial sources and used without further purification. The raw materials are shown as follows: Poly vinyl alcohol (PVA, 1799, Alcoholysis degree: 98～99% (mol/mol)), $K_3Fe(CN)_6$ (*MW* = 329.25, ≥ 99.5%, subsequently abbreviated as $Fe(CN)_6^{3-}$), $K_4Fe(CN)_6·3H_2O$ (*MW* = 422.39, 99.0%, subsequently abbreviated as $Fe(CN)_6^{4-}$) were purchased from Aladdin Industrial Corporation. KCl (*MW* = 74.55, 99.8%) were provided by Macklin Biochemical CO., Ltd. Pyrrole (*MW* = 67.09, 99%), 4-Acetylphenylboronic acid (*MW* = 163.97, 98%) and methane sulfonic acid (*MW* = 96.11, 99%) were provided by Energy Chemical. Tetrabutylammonium chloride (TBA-Cl, 97%) was provided by Shanghai yuanye Bio-Technology Co., Ltd.

Method of simulation calculation. We used the DFT as implemented in the Vienna Ab initio simulation package (VASP) in all calculations. The exchange-correlation potential is described by using the generalized gradient approximation of Perdew-Burke-Ernzerhof (GGA-PBE). The projector augmented-wave (PAW) method is employed to treat interactions between ion cores and valence electrons. The plane-wave cutoff energy was fixed to 500 eV. Given structural models were relaxed until the Hellmann–Feynman forces smaller than -0.02 eV/Å and the change in energy smaller than 10^{-5} eV was attained. During the relaxation, the Brillouin zone was represented by a Γ centered k-point grid of 10×10×10. Grimme's DFT-D3 methodology was used to describe the dispersion interactions among all the atoms in adsorption models.

Application demonstration of TECs. The respiration monitoring device consists of three 1 × 3 × 0.5 cm TECs connected in series with conductive copper tape and encapsulated with PI tape. The touch interaction device is composed of three 1 × 1 × 1 cm TECs connected in series using the same method. The TECs array is made up of nine 2 × 2 × 1 cm TECs units, connected in series by copper strips in a 10 × 10 cm acrylic mold and finally encapsulated with PI tape. Four of these arrays are connected in series to obtain a large device with 36 TECs units. The TEC materials used for all the above TECs were PVA/C4P/KCl/$Fe(CN)_6^{3-/4-}$ (C4P: 1 mM, KCl: 0.1 M, $Fe(CN)_6^{3-/4-}$: 0.3 M, PVA: 10 wt%). One side of the device collects thermal energy from the human body or a heated platform, while the other side is exposed to air to create a temperature difference.

Interactive Signal Encoding and Wireless Transmission. To demonstrate the practical applicability of the TEC array as a self-powered signal source, an interactive communication system was constructed. Three TEC units were connected in series and interfaced with an Arduino Uno board, where the open-circuit voltage generated under a temperature gradient served as the input signal. Custom firmware continuously monitored the voltage and classified it into three discrete levels, each corresponding to a predefined text message, which was then wirelessly transmitted to a smartphone via an HC-05 Bluetooth module.

Supplementary Discussion

Calculation of the thermopower. Due to the introduction of KCl, both thermodiffusion and thermoelectric effects occur within the TECs gel. The synergistic effects of thermodiffusion and thermoelectric effects are discussed in detail in Han's previous

reported work and will not be reiterated here(*1*). The thermopower is calculated according to Equation 1:

$$S_e = S_{tg} + S_{td} \tag{1}$$

where S_e is the thermopower of the TEC, S_{td} refers to the thermopower provided by the thermodiffusion effect, and S_{tg} is the thermopower provided by the thermogalvanic effect.

The thermopower of thermodiffusion can be interpreted as the entropy transferred by the thermodiffusion of ions, and the thermopower of thermodiffusion is defined as the ratio between the electric field - dV/dx and the temperature gradient dT/dx, which can be written as:

$$S_{td} = -\frac{dV/dx}{dT/dx} = -\frac{V(T_H)-V(T_C)}{T_H-T_C} \tag{2}$$

where S_{td} is the thermodiffusion thermopower, V is the voltage, and T_H and T_C are the temperatures of the hot and cold electrodes, respectively. Equation (2) indicates that for p-type thermodiffusion thermopower, the voltage is negative when the positive electrode is attached to hot end and the voltage is positive when the negative electrode is attached to hot end.

The thermopower provided by the thermoelectric effect can be considered as the final thermally induced voltage resulting from the redox contribution to the temperature-dependent standard electrode potential E_0(*2*). Thus, the thermopower measured by the thermoelectric effect in the presence of a temperature difference can be written as:

$$S_{tg} = -\frac{E^0(T_H)-E^0(T_C)}{T_H-T_C} \approx -\frac{V(T_H)-V(T_C)}{T_H-T_C} \tag{3}$$

In Equation (3), S_{tg} is the thermopower provided by the thermoelectric effect, $E^0(T_H/T_C)$ represents the electrode potentials shown at the hot and cold ends. In the absence of thermodiffusion involved, it is measured in the form of voltage.

As can be seen from Eqs.1~3, the thermopower of the TECs can ultimately be expressed as the ratio of the voltage difference between the hot and cold ends to the temperature difference. Therefore, the following equation can be used to calculate the thermopower of this synergistic effect:

$$S_e = S_{tg} + S_{td} = -\frac{V(T_H)-V(T_C)}{T_H-T_C} \quad (4)$$

In this work, we use "thermopower" as a generic term to describe the thermal pressure difference induced by either the thermodiffusion effect or the thermogalvanic effect, and to elucidate the notational conventions for these two different mechanisms.

Calculation of the normalized output power density ($P_{max}/\Delta T^2$). For the normalized maximum power density tests, the distance between the two electrodes was set to 1 cm, and the cross-sectional area of the thermogalvanic cell was 1 cm^2. The current-voltage curves were measured from 0 V to the open-circuit voltage. The power-voltage curves were calculated according to Eq. 5(*3*):

$$P_{max} = \frac{V_{oc}I_{sc}}{4} \quad (5)$$

where V_{oc} and I_{sc} are the open-circuit voltage and short-circuit current, respectively.

Calculation of Carnot-relative efficiency (η_r) and figure of merit (ZT). The energy conversion efficiency (η) of a thermoelectric device is defined as the ratio of the maximum electrical output power (P_{max}) from the thermogalvanic cell to the heat input power (P_{heat}).

$$\eta = \frac{P_{max}}{P_{heat}} = \frac{P_{max}d}{\kappa\Delta T} \quad (6)$$

where d, ΔT, and κ are the distance, temperature difference between hot and cold electrodes and thermal conductivity respectively.

It is worth noting that the S_{td} provided by thermodiffusion in TECs is capacitance-like, and part of its energy is rapidly dissipated in the presence of an external circuit, retaining only the energy provided by the thermogalvanic effect. Therefore, a reconstruction of P_{max} is required when measuring the thermal conversion efficiency of TECs, retaining only the thermopower associated with the thermogalvanic effect.

$$P_{tg} = \frac{V_{oc}I_{sc}}{4} = \frac{S_{tg}\Delta T \cdot \sigma V_{oc}}{4d} = \frac{S_{tg}{}^2 \cdot \sigma}{4d} \cdot \Delta T^2 \quad (7)$$

where P_{te} and σ are the power and conductivity of TECs under the thermoelectric effect, respectively. The conductivity was obtained by measuring the voltage-current curve with a Keithley 2450 system. The detailed conductivity is shown in Supplementary Fig 8.

$$\eta = \frac{P_{tg}}{P_{heat}} = \frac{P_{tg}d}{\kappa\Delta T} = \frac{S_{tg}{}^2\sigma\Delta T}{4\kappa} \quad (8)$$

In the above calculations, S_{tg} was 4.5 mV/K, σ was 3.25 S/m, κ was 0.36 W/m·K, d was 1 cm, and ΔT was 10 K. Calculations show that the thermoelectric conversion efficiency (η) of the TECs at 10 K temperature difference is 0.046%.

$$\eta_r = \frac{\eta \cdot T_{hot}}{\Delta T} = \frac{P_{tg}d \cdot T_{hot}}{\kappa\Delta T^2} \quad (9)$$

where T_{hot} is the temperature at the hot side of the sample. The value of T_{hot} was 308.15 K. The Carnot-relative efficiency (η_r) is 1.42%.

The performance of thermogalvanic cells is typically evaluated using the figure-of-merit (ZT= $S_e^2\sigma/k$). However, this definition is not directly applicable to the cells

with thermodiffusion effects due to their transient behavior. In the current work, we observed that the thermodiffusion effect has a minor impact on the thermopower. Therefore, we amended the calculation of the ZT by subtracting the thermodiffusion contribution factor, which is often calculated according to Eq. 10.

$$ZT = \frac{(S_e - S_{td})^2 \sigma T}{\kappa} = \frac{{S_{tg}}^2 \sigma T}{\kappa} \tag{10}$$

where T is the average temperature of the hot and cold ends of the TECs, T was 303.15 K. The ZT of TECs is 0.06.

Mechanism of enhancement of thermoelectric effect by C4P. The enhancement of thermopower in our dual-ion regulated gel-based thermoelectric cells arises from the synergistic interplay between selective host–guest complexation and thermally induced concentration gradients. The C4P macrocycle selectively binds anionic species, particularly $Fe(CN)_6^{4-}$, and this interaction plays a pivotal role in modulating both the entropy change and ionic transport asymmetry across a thermal gradient. In ionic thermogalvanic cells, the thermopower (S_{tg}) arises from the difference in redox potential between hot and cold electrodes, which is governed by entropy differences and concentration distributions of the redox-active ions. According to the extended Nernst-based formulation as shown in previous reported studies (*19*):

$$S_{tg} = \frac{R}{nF\Delta T}\left[T_{hot}\ln\frac{(\gamma A)^a_{hot}}{(\gamma B)^b_{hot}} - T_{cold}\ln\frac{(\gamma A)^a_{cold}}{(\gamma B)^b_{cold}}\right] + \frac{R}{nF\Delta T}\left[T_{hot}\ln\frac{[A]^a_{hot}}{[B]^b_{hot}} - T_{cold}\ln\frac{[A]^a_{cold}}{[B]^b_{cold}}\right] \tag{11}$$

In the formula, n represents the number of electrons transferred in the redox reaction, and F is the Faraday constant. γ is the activity coefficient (related to solvation and entropy), and A and B represent the redox ions. In this study, A is $Fe(CN)_6^{4-}$, and B is $Fe(CN)_6^{3-}$. This equation reveals that the Seebeck coefficient has two main

contributions: 1) Entropy-Driven Term (activity-based): Sensitive to changes in solvation environment and ion pairing; 2) Concentration-Driven Term: Arises from temperature-dependent concentration gradients of the redox ions.

C4P influences both components simultaneously, as detailed below.

C4P-Induced Enhancement of Redox Entropy Contribution. $Fe(CN)_6^{4-}$ is a highly charged, strongly hydrated anion. Upon forming a host–guest complex with C4P, its local solvation shell becomes disrupted, and the degrees of freedom of the anion are significantly reduced. This has two key consequences. At the cold end (lower temperature): The host–guest complexation between C4P and $Fe(CN)_6^{4-}$ is favored, due to its exothermic binding enthalpy (a well-known characteristic of anion recognition in macrocyclic hosts). As a result, a significant fraction of $Fe(CN)_6^{4-}$ exists in a bound and electrochemically inactive state. At the hot end (higher temperature): Thermal energy disrupts the host–guest interaction, increasing the proportion of free, unbound $Fe(CN)_6^{4-}$. This difference in chemical speciation leads to an asymmetric entropy change between the hot and cold sides during the redox reaction:

$$Fe(CN)_6^{4-} \rightleftharpoons Fe(CN)_6^{3-} + e^{-} \quad (12)$$

As the electrochemical activity of $Fe(CN)_6^{4-}$ is suppressed at the cold side but retained at the hot side, the entropy change per electron transferred (ΔS) increases, directly enhancing the thermopower via:

$$S_{redox} = \frac{\Delta S}{nF} \quad (13)$$

This was validated experimentally by CV, where the addition of C4P caused a notable decrease in redox current, indicating reduced electrochemical activity due to complexation (Supplementary Fig 26). The entropy loss arises from host–guest dissociation upon oxidation at the hot side, and the re-formation of complexes upon reduction at the cold side, both steps contributing to greater disorder (positive entropy change).

C4P-Modulated Redox Ion Concentration Gradients. In addition to modulating entropy, C4P also significantly affects the temperature-dependent concentration gradient of free

$Fe(CN)_6^{4-}$ ions across the cell. The binding equilibrium can be described as:

$$K_{as} = \frac{[C4P/FeCN^{4-}]}{[C4P][FeCN^{4-}]} \quad (14)$$

Combined with mass balance equations:

$$[C4P]_0 = [C4P] + [C4P/FeCN^{4-}] \quad (15)$$

$$[FeCN^{4-}]_0 = [FeCN^{4-}] + [C4P/FeCN^{4-}] \quad (16)$$

K_{as} is the complexation constant, [X] is the concentration of the corresponding material, and $[X]_0$ is the initial concentration of the material. According to the above formulas, We derive the temperature-dependent free ion concentration:

$$[FeCN^{4-}]=\frac{1}{2}\left\{[FeCN^{4-}]_0-[C4P]_0-K_{as}^{-1}+\sqrt{\left([FeCN^{4-}]_0+[C4P]_0+K_{as}^{-1}\right)^2-4[C4P]_0[FeCN^{4-}]_0}\right\} \quad (17)$$

Our experimental data show that K_{as} decreases with increasing temperature, confirming weaker binding at the hot side (Supplementary Fig 14). This leads to a higher concentration of free $Fe(CN)_6^{4-}$ at the hot end, and a lower concentration at the cold end, thereby establishing a redox species concentration gradient that further contributes to S_e.

Thus, the concentration-driven term:

$$S_{conc} = \frac{R}{nF\Delta T}\left[T_{hot}\ln\frac{[A]_{hot}^{a}}{[B]_{hot}^{b}} - T_{cold}\ln\frac{[A]_{cold}^{a}}{[B]_{cold}^{b}}\right] \quad (18)$$

is also amplified, resulting in an overall increase in total thermopower.

The significant enhancement in the thermopower arises from the dual effect of C4P: it increases redox entropy through complexation/dissociation dynamics and enhances ion concentration gradients via its temperature-sensitive binding equilibrium. These two mechanisms work synergistically to boost thermopower. Experimentally, the thermopower increased from ~1.4 mV/K (without C4P) to ~4.8 mV/K (with C4P), accompanied by a corresponding rise in normalized power density. The observed improvement stems directly from molecular-level interactions between C4P and $Fe(CN)_6^{4-}$, modulated by temperature.

Supplementary Figures

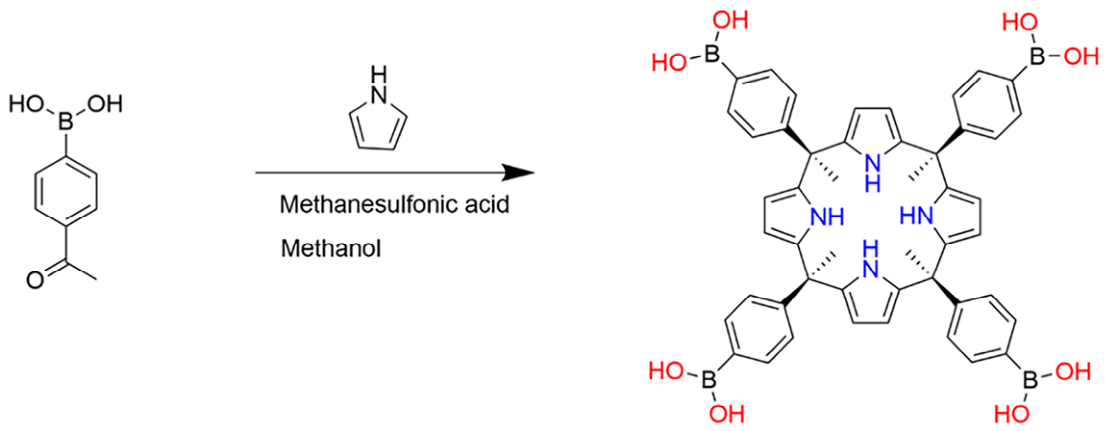

Supplementary Fig 1. Synthesis of C4P.

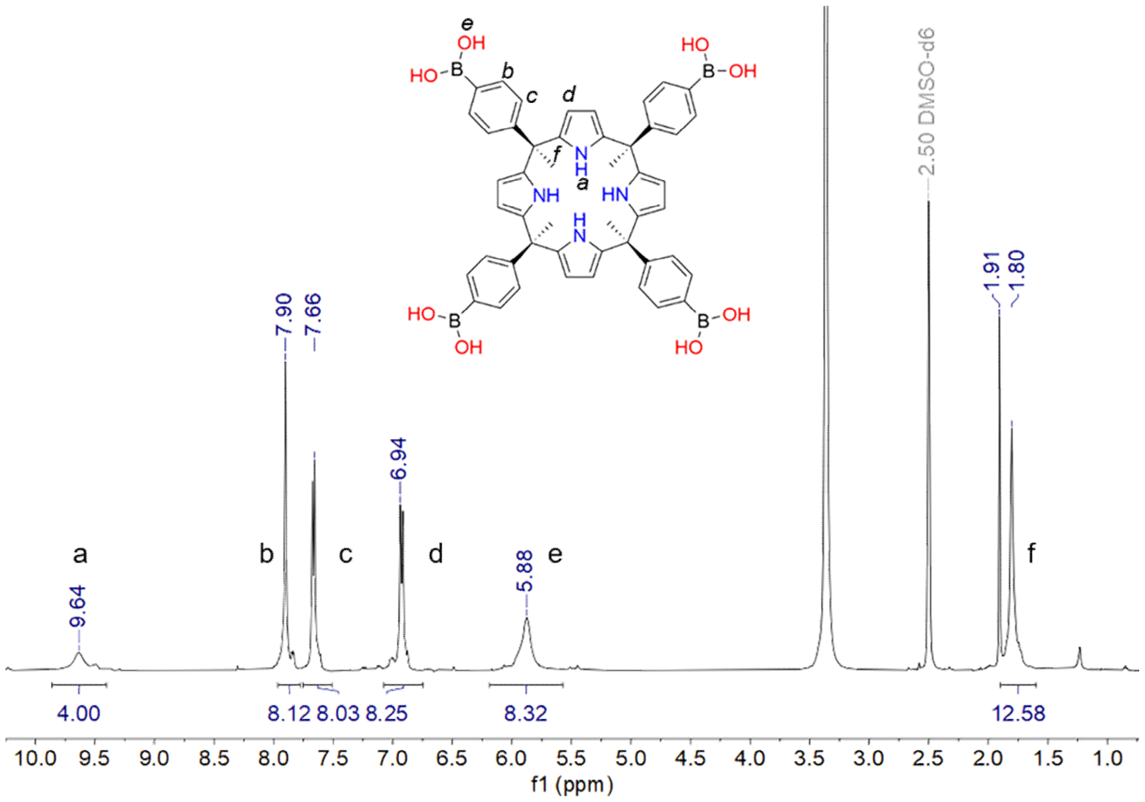

Supplementary Fig 2. ^{1}H NMR spectrum (400 MHz, DMSO-d_6, 25 °C) of C4P. The peaks labeled a–f are assigned to protons at distinct positions in the C4P molecular structure.

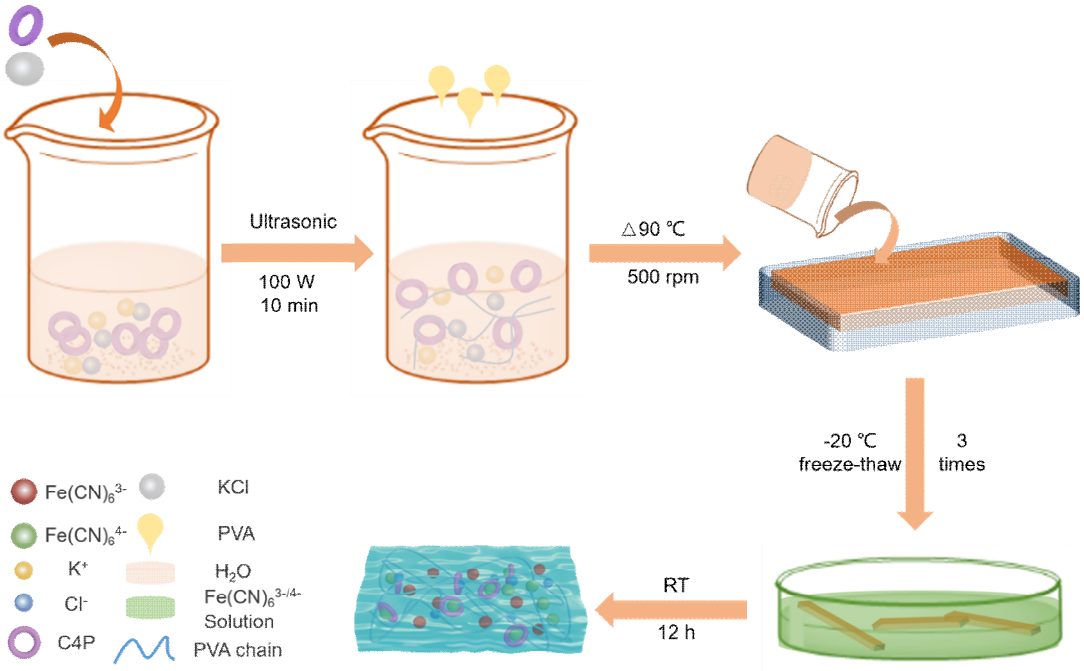

Supplementary Fig 3. Illustration of the preparation process of TECs gel materials.

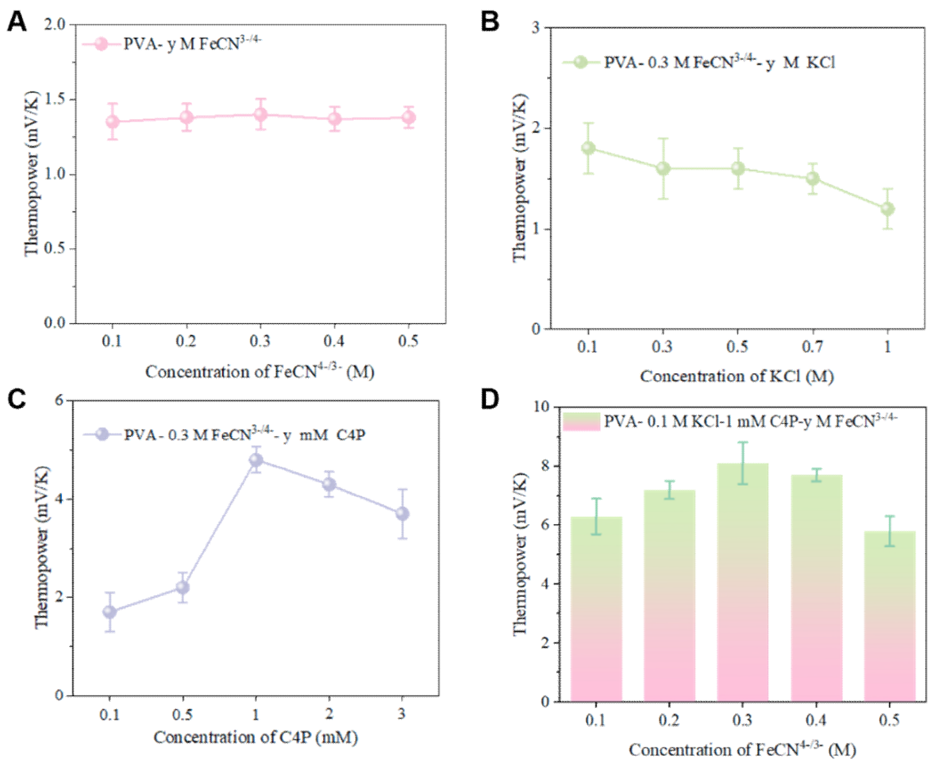

Supplementary Fig 4. Performance optimization of TECs. Thermoelectric performance of the TECs measured at different concentrations of (A) $Fe(CN)_6^{3-/4-}$, (B) KCl and (C) C4P, respectively. (D) Thermoelectric performance as a function of $Fe(CN)_6^{3-/4-}$ concentration, while keeping the concentrations of C4P and KCl constant. The optimal concentrations were found to be 0.3 M for $Fe(CN)_6^{3-/4-}$, 0.1 M for KCl, and 1 mM for C4P. Considering the potential for synergistic effects among these components, we further tested the thermoelectric power of the gel by immersing it in different concentrations of $Fe(CN)_6^{3-/4-}$ solution after determining the optimal amount of KCl and C4P. The TECs contained 0.3 M $Fe(CN)_6^{3-/4-}$ exhibited the best performance. Data are presented as mean values ± SD (n = 3).

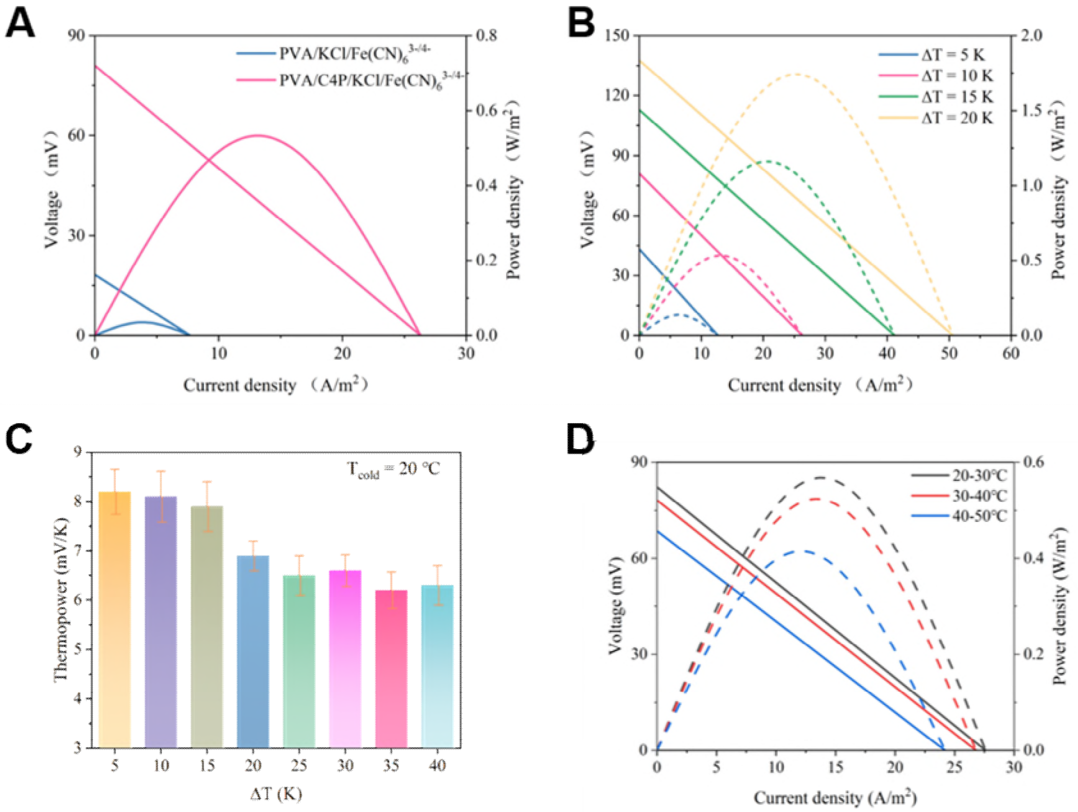

Supplementary Fig 5. Performance of the gel under different conditions. (A) Current-voltage curves and corresponding power densities for PVA/KCl/Fe(CN)$_6^{3−/4−}$ and PVA/C4P/KCl/Fe(CN)$_6^{3−4−}$ at a temperature difference of 10 K. (B) Current-voltage curves and corresponding power densities for PVA/C4P/KCl/Fe(CN)$_6^{3−/4−}$ under different temperature differences. (C) Thermopower variation of PVA/C4P/KCl/Fe(CN)$_6^{3−/4−}$ under different temperature differences. Data are presented as mean values ± SD (n = 3). (D) I–V curves of PVA/C4P/KCl/Fe(CN)$_6^{3−/4−}$ across different temperature ranges.

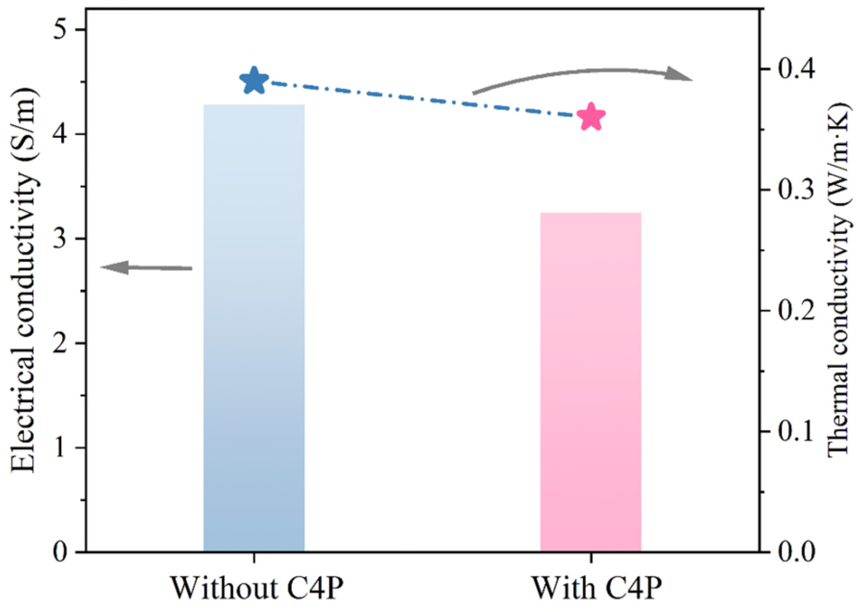

Supplementary Fig 6. The electrical conductivities and thermal conductivities of PVA/KCl/$Fe(CN)_6^{3-/4-}$ and PVA/C4P/KCl/$Fe(CN)_6^{3-/4-}$. The conductivity was calculated from the I-V curve.

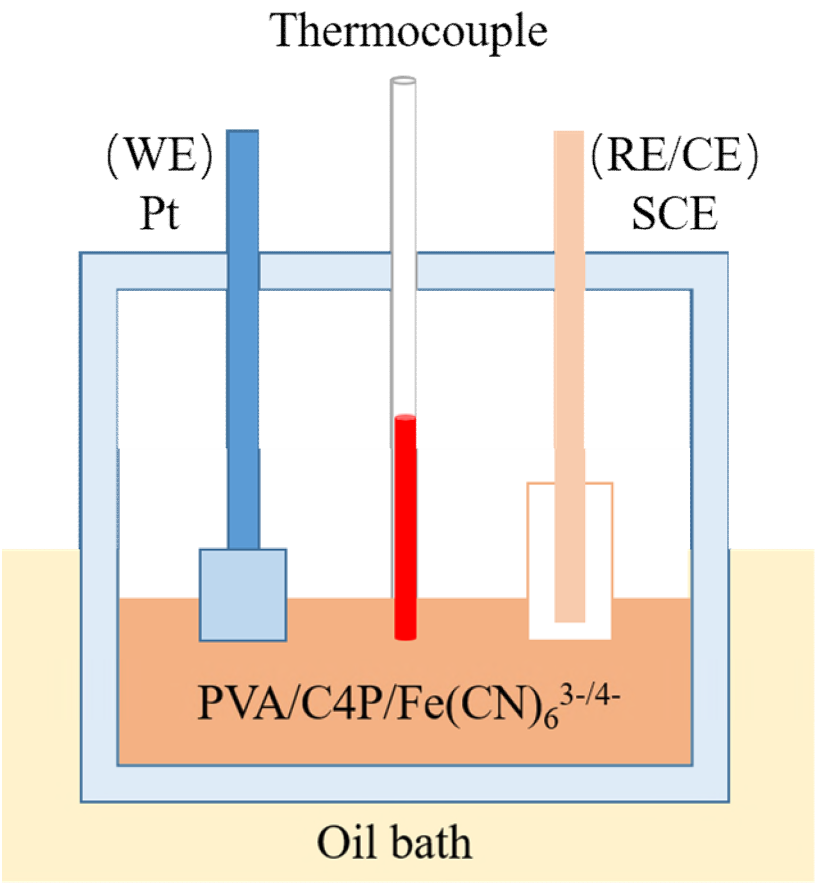

Supplementary Fig 7. Isothermal system of PVA-$Fe(CN)_6^{4-/3-}$ for measuring the entropy of $Fe(CN)_6^{4-/3-}$. The work electrode (WE) was platinum, whereas SCE was used as the reference electrode (RE) and counter electrode (CE).

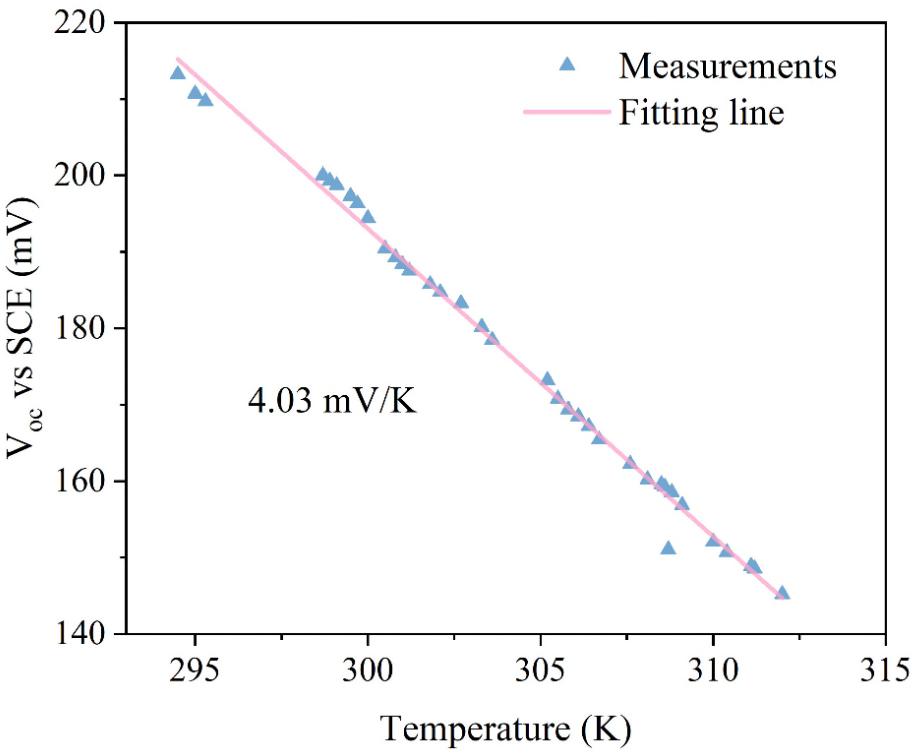

Supplementary Fig 8. Open-circuit voltage vs. SCE (saturated calomel electrode) with the dependent of temperature in an isothermal three-electrode system. Temperature coefficient relative to SCE extracted by linear fitting is −4.03 mV/K vs. SCE. Note that the SCE itself has a temperature coefficient of −0.47 mV/K, therefore the temperature coefficient of $Fe(CN)_6{}^{4-/3-}$ is −4.5 mV/K.

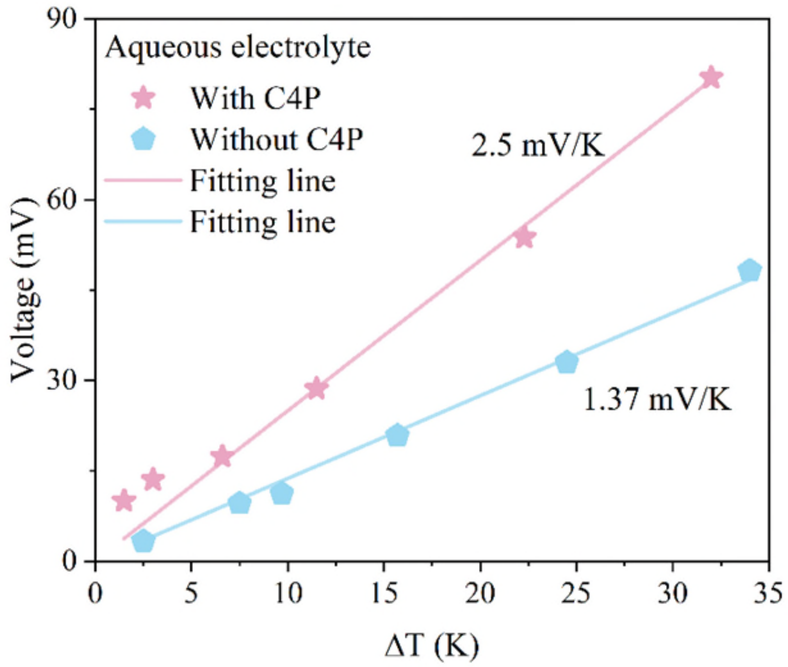

Supplementary Fig 9. The voltage change images of thermoelectric batteries before and after the addition of C4P in aqueous electrolytes were obtained, and the thermopower was determined by linear fitting. The ion concentration and C4P addition amount were consistent with those of gel batteries.

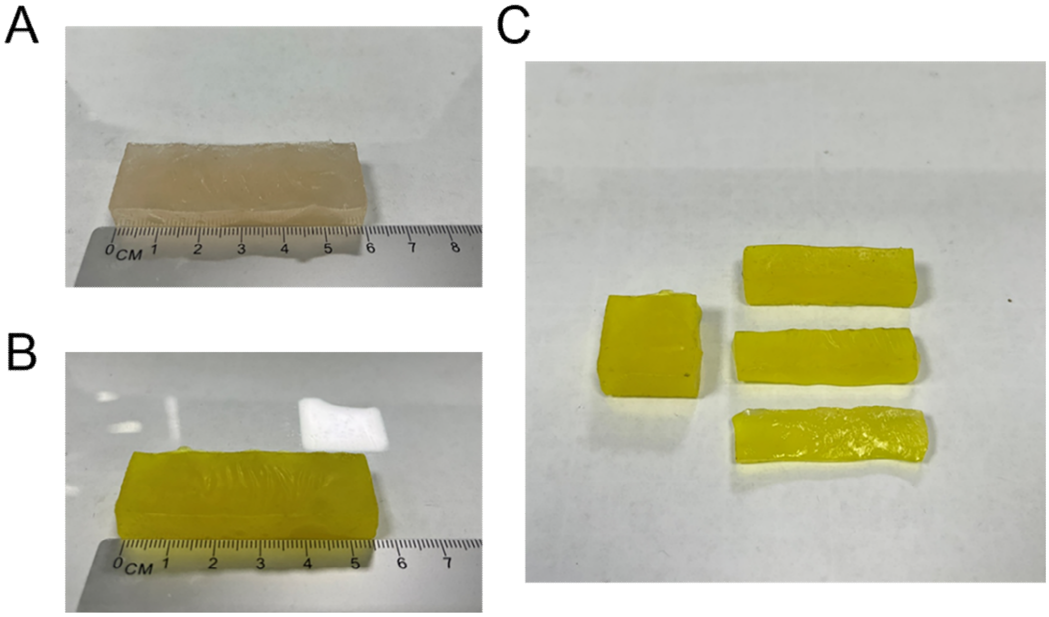

Supplementary Fig 10. Display image of the gel. (A) PVA/C4P/KCl gel. (B) PVA/C4P/KCl/ $Fe(CN)_6^{3-/4-}$ gel. (C) The gel cutted into different shapes with scissors. The dimensions of the gel were 2 × 6 × 1 cm. After immersion in the $Fe(CN)_6^{3-/4-}$ solution, the gel exhibited some morphological changes, with a noticeable shortening of approximately 0.5 cm in length, as shown in (B). This change is attributed to the solvent substitution between the $Fe(CN)_6^{3-/4-}$ solution and the gel, which unavoidably induced cross-linking of the PVA molecular chains, leading to the observed morphological alteration.

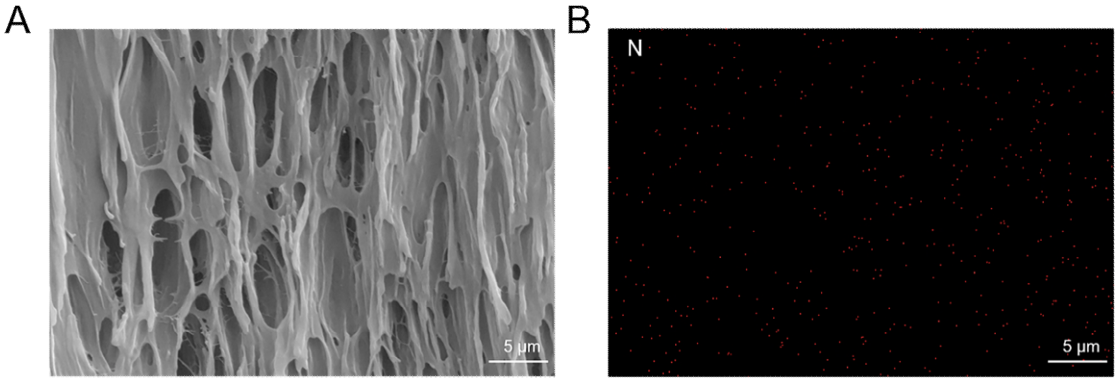

Supplementary Fig 11. Cross-sectional SEM and EDX mapping of the gel. (A) Cross sectional SEM of the gel. (B) Corresponding EDX mapping of gel. The sample contained 1 mM of C4P, resulting in fewer atomic spots of element N detected in the EDX mapping.

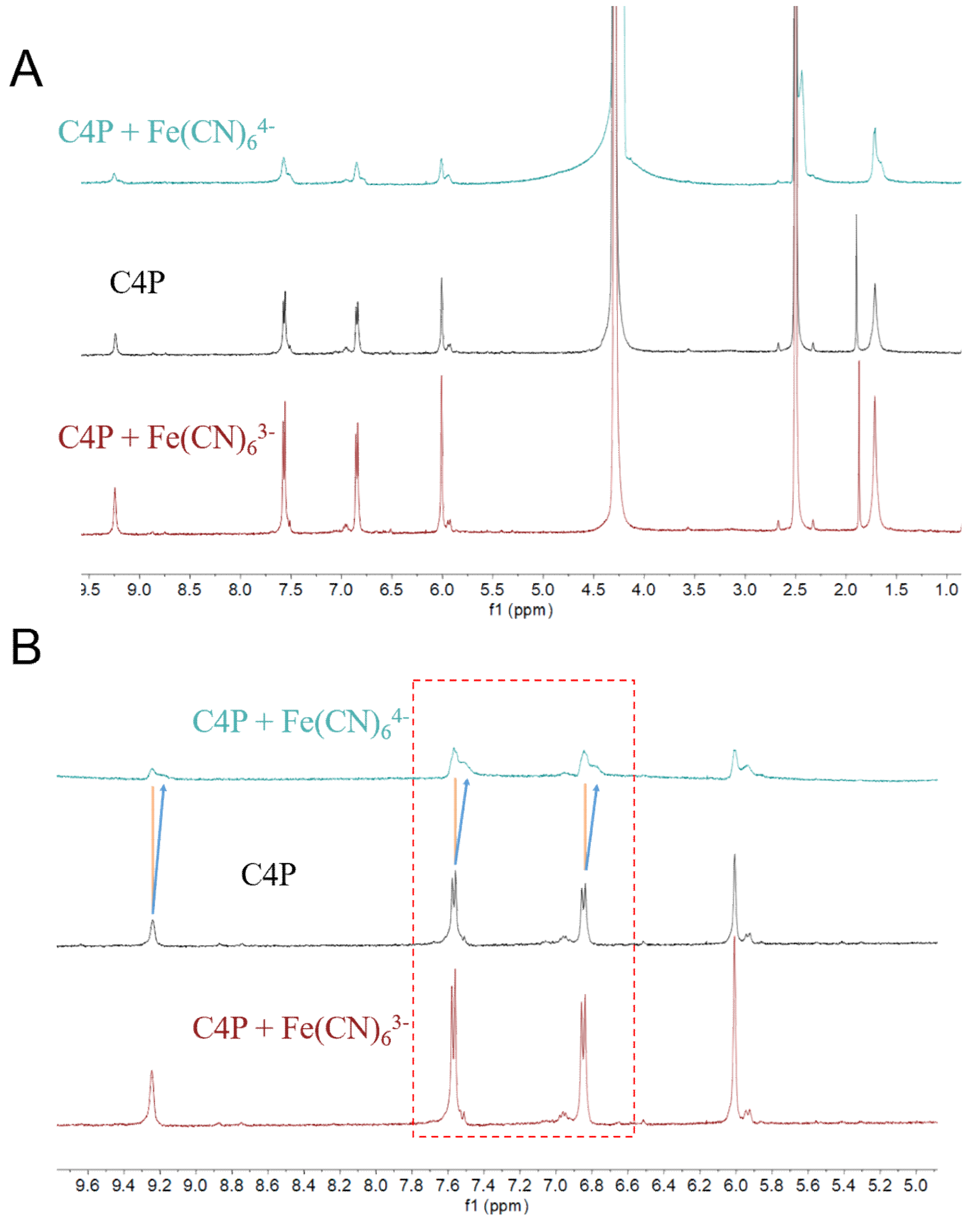

Supplementary Fig 12. ^{1}H NMR profiles (400 MHz, DMSO-d_6: D_2O = 9:1, 25 °C) of C4P, C4P/$Fe(CN)_6^{3-}$ and C4P/$Fe(CN)_6^{4-}$. (A) Full spectrum. (B) Enlarged region from 5.0 to 9.6 ppm.

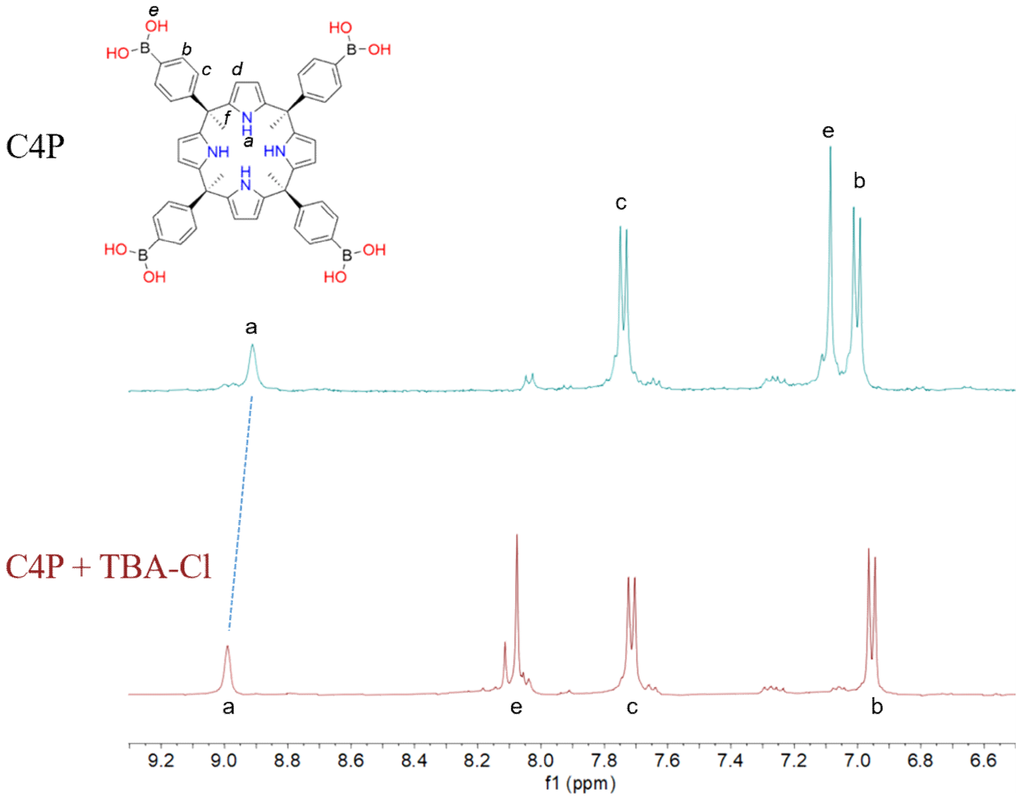

Supplementary Fig 13. ^{1}H NMR spectra (400 MHz, 298 K, acetone-d_6) of C4P and C4P/TBACl. A pronounced downfield shift of the H_a proton is observed upon addition of TBACl, indicating the formation of a host–guest complex between C4P and Cl^-.

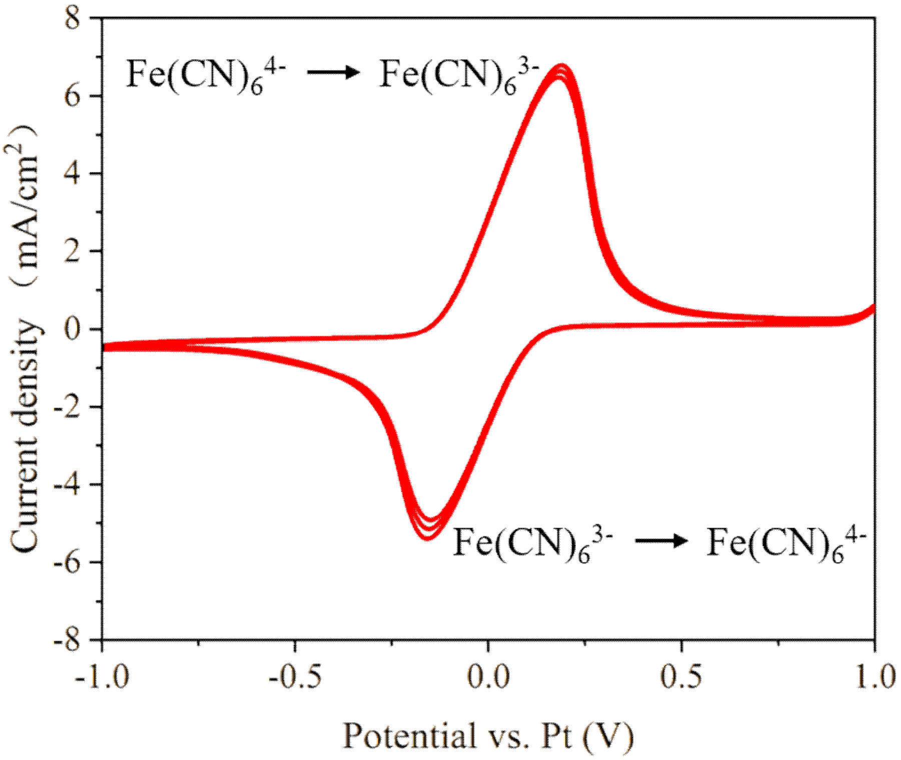

Supplementary Fig 14. CV curves of PVA/C4P/KCl/$Fe(CN)_6^{3-/4-}$. The sample was sandwiched between two 0.5×0.5 cm platinum sheets, and the scanning speed was set to 100 mV/s, with a total of three scanning circle.

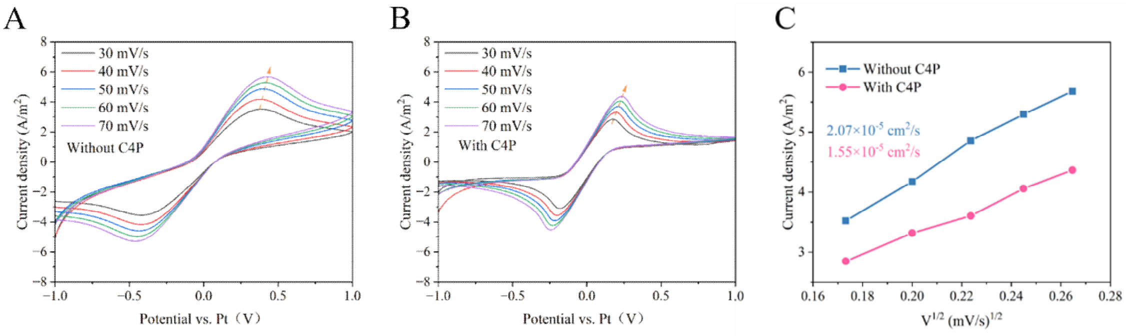

Supplementary Fig 15. Cyclic voltammetry (CV) curves of the gel at varying sweep rates (A) before and (B) after C4P incorporation. (C) Ion mobility values derived from the CV data, showing the effect of C4P on ionic transport within the gel.

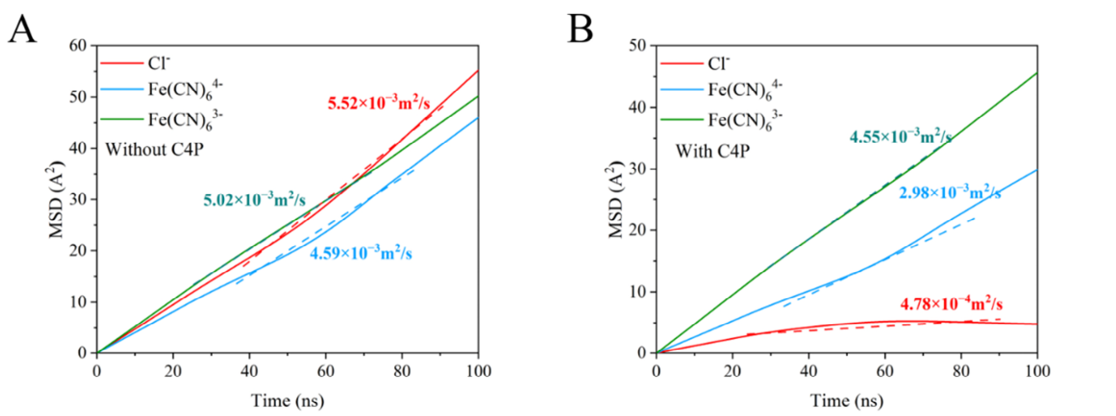

Supplementary Fig 16. Molecular dynamics simulation of ionic mobility. (A) Simulated mobility of ions before interaction with C4P. (B) Ion mobility after the addition of three ions to C4P.

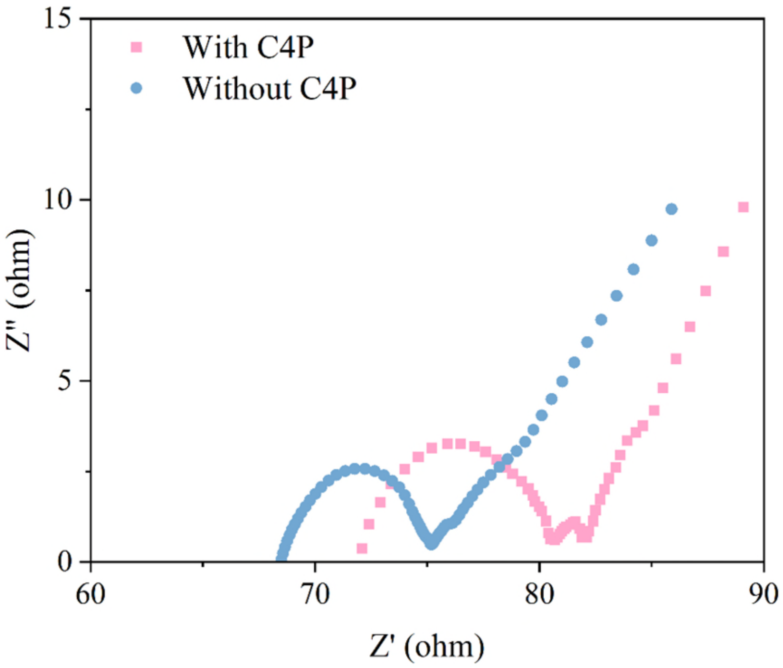

Supplementary Fig 17. Electrochemical impedance spectroscopy (EIS) for PVA/KCl/$Fe(CN)_6^{3-/4-}$ and PVA/C4P/KCl/$Fe(CN)_6^{3-4-}$. The electrode separation distance (L) is 1 cm, and the electrode area is 0.25 cm^2. With the addition of C4P, the calculated conductivity of the device is 4.12 S/m.

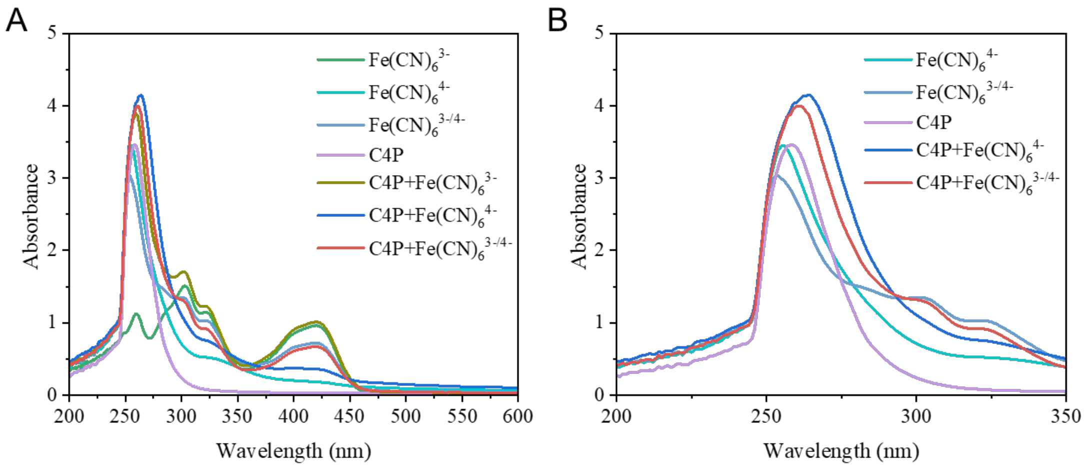

Supplementary Fig 18. UV-Vis spectra of C4P and $Fe(CN)_6^{3-/4-}$ systems. (A) UV–vis spectra of $Fe(CN)_6^{3-}$, $Fe(CN)_6^{4-}$, C4P, and their mixtures. (B) UV–vis spectra of $Fe(CN)_6^{4-}$, $Fe(CN)_6^{3-/4-}$, C4P, and their corresponding mixtures with C4P. The characteristic peak of $Fe(CN)_6^{4-}$ is located at 256 nm, while the characteristic peak of C4P is 258 nm. After mixing the two, a single higher peak appears in the spectra at 264 nm. This indicates that C4P can bind $Fe(CN)_6^{4-}$, resulting in the merging of their peaks and a significant chemical shift of 8 nm relative to $Fe(CN)_6^{4-}$. However, when we mixed C4P with a solution containing both $Fe(CN)_6^{3-}$ and $Fe(CN)_6^{4-}$, the signal peaks corresponding to $Fe(CN)_6^{4-}$ showed the same behavior as in the single component mixture. Meanwhile, the addition of C4P has no effect on the signal peak of $Fe(CN)_6^{3-}$, indicating no host-guest between C4P and $Fe(CN)_6^{3-}$.

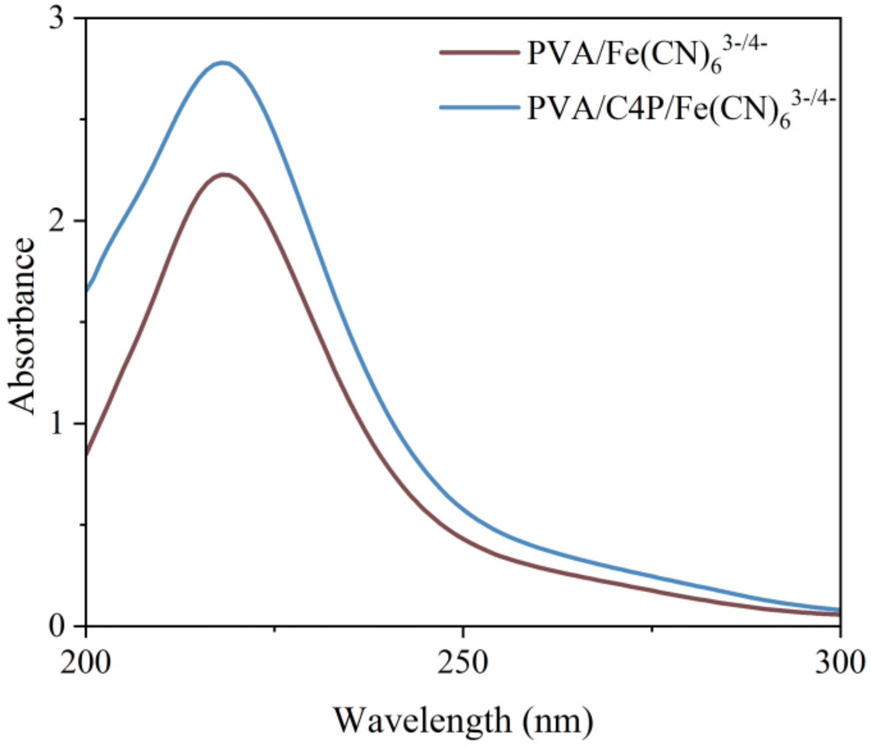

Supplementary Fig 19. UV-Vis spectra of C4P/Fe(CN)$_6^{3-/4-}$ in the presence of PVA, demonstrating that C4P maintains its complexation with Fe(CN)$_4^-$ within the PVA gel matrix.

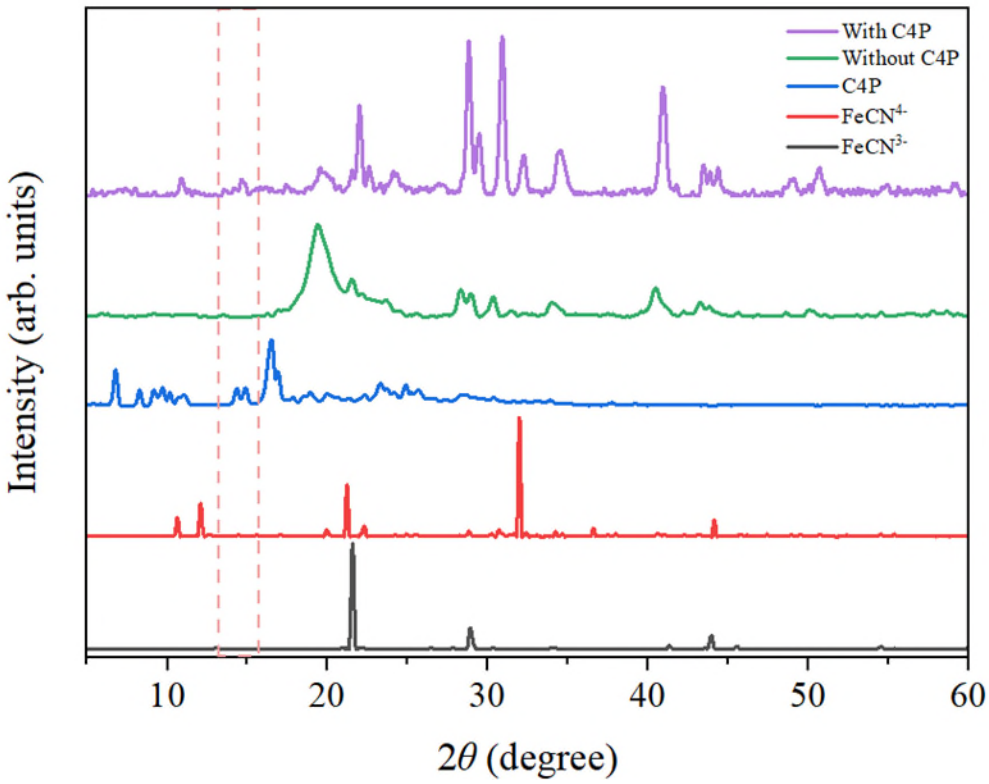

Supplementary Fig 20. XRD pattern of TECs and their individual components. Due to the relatively weak host-guest complexation between C4P and $Fe(CN)_6^{4-}$, the characteristic peaks of $Fe(CN)_6^{4-}$ remain visible even after drying the gel. A representative diffraction peak corresponding to C4P is highlighted in the red box.

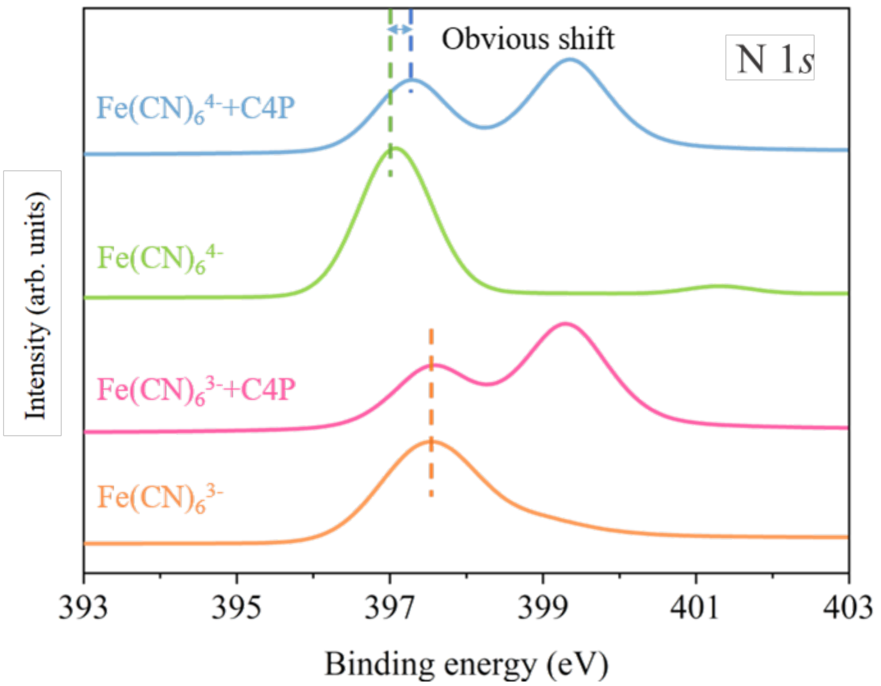

Supplementary Fig 21. XPS N 1*s* spectra of $Fe(CN)_6^{3-}$, C4P/$Fe(CN)_6^{3-}$ and $Fe(CN)_6^{4-}$, C4P/$Fe(CN)_6^{4-}$.

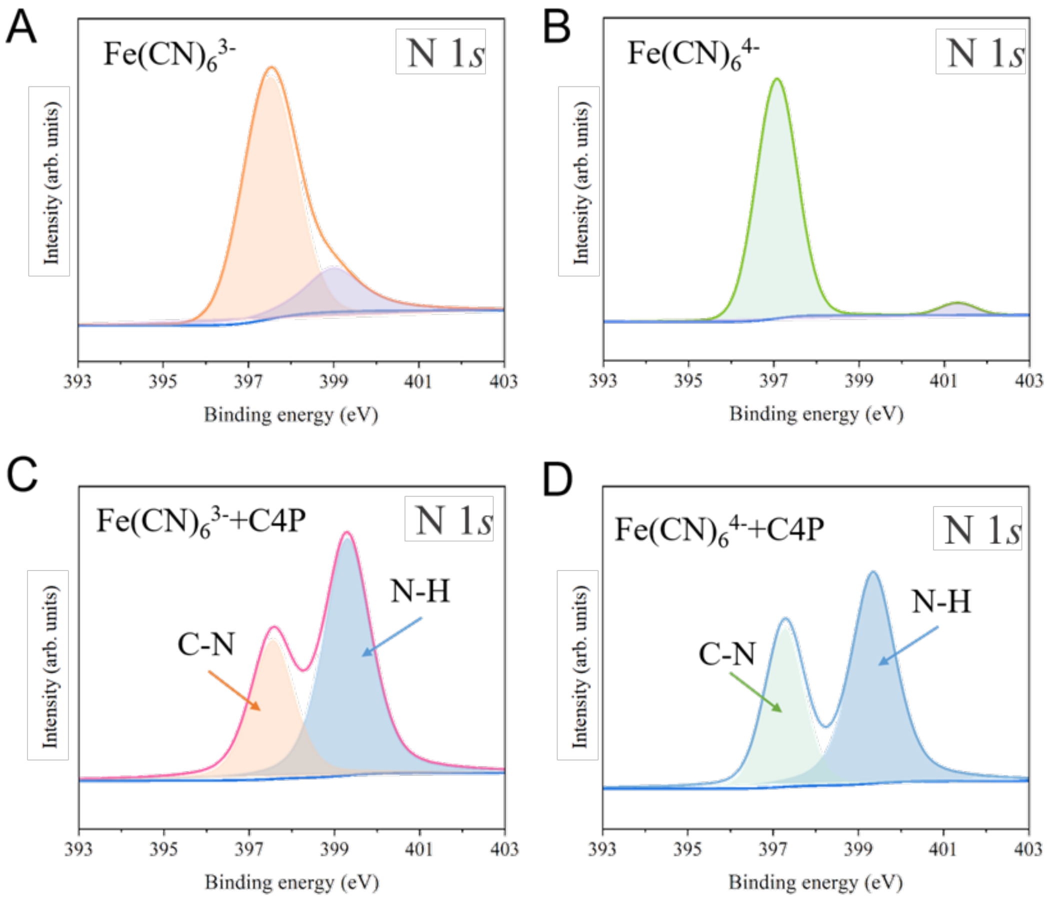

Supplementary Fig 22. N 1*s* XPS spectra of (A) $Fe(CN)_6^{3-}$. (B) $Fe(CN)_6^{4-}$. (C) $Fe(CN)_6^{3-}$+C4P. (D) $Fe(CN)_6^{4-}$+ C4P. Solid composite samples for XPS measurements were prepared by drying the composite solution in a 333 K vacuum oven for 24 hours.

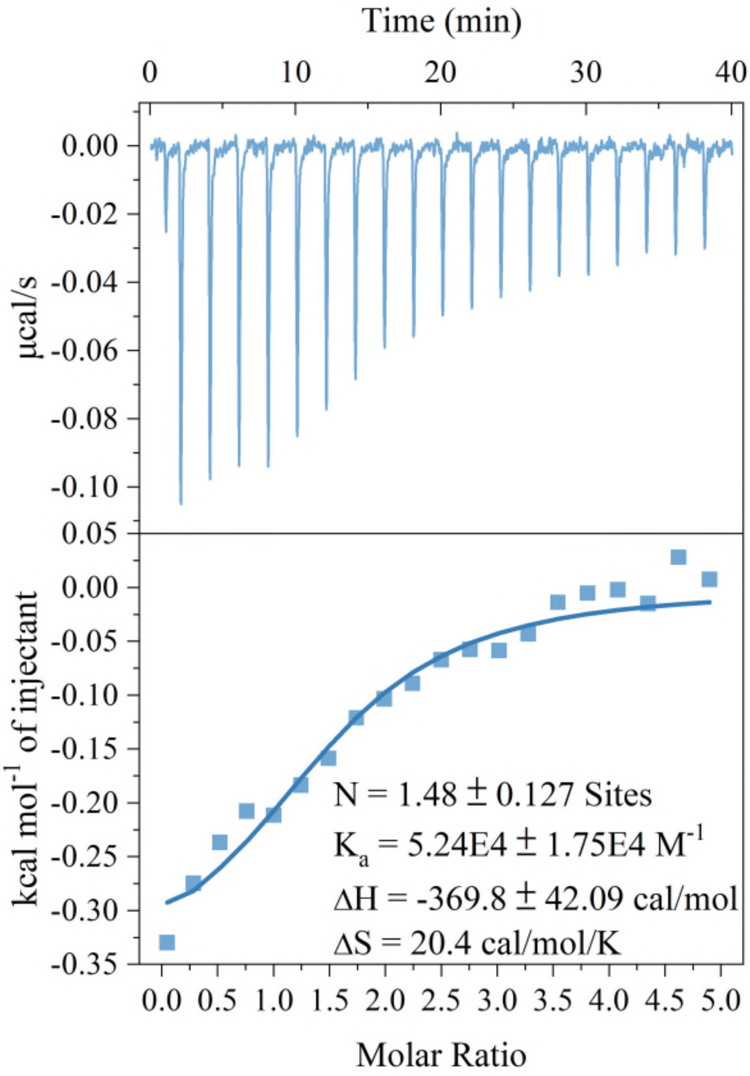

Supplementary Fig 23. Isothermal titration calorimetry analysis of the complexation between C4P and $Fe(CN)_6^{4-}$. The titration was performed at 25 °C by incrementally adding C4P into an aqueous solution of $Fe(CN)_6^{4-}$.

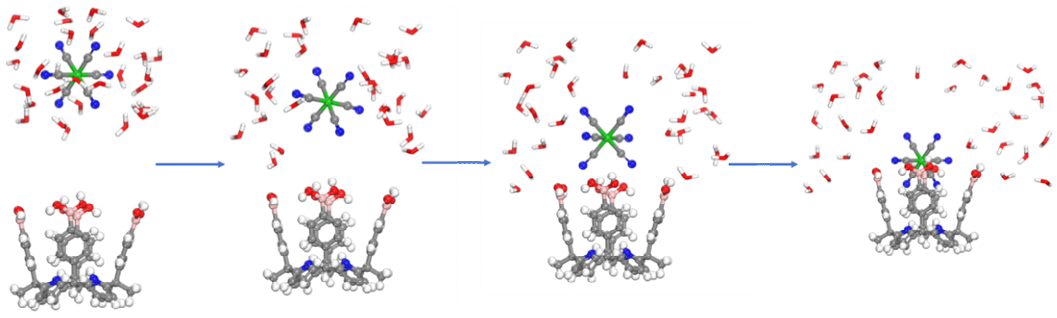

Supplementary Fig 24. Molecular dynamics simulation of the complexation process between C4P and $Fe(CN)_6^{4-}$.

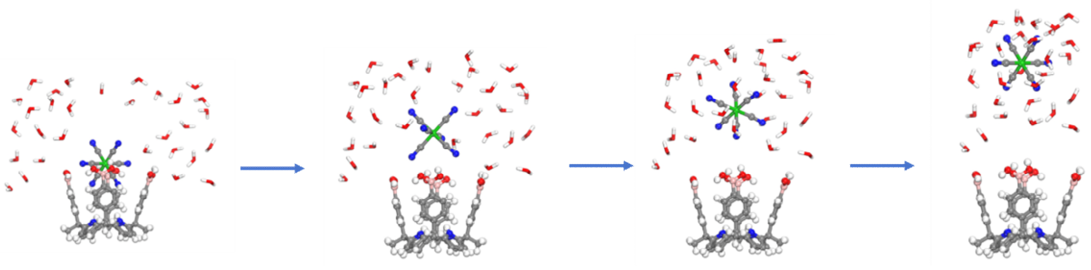

Supplementary Fig 25. Molecular dynamics simulation of the thermal dissociation process between C4P and $Fe(CN)_6^{4-}$.

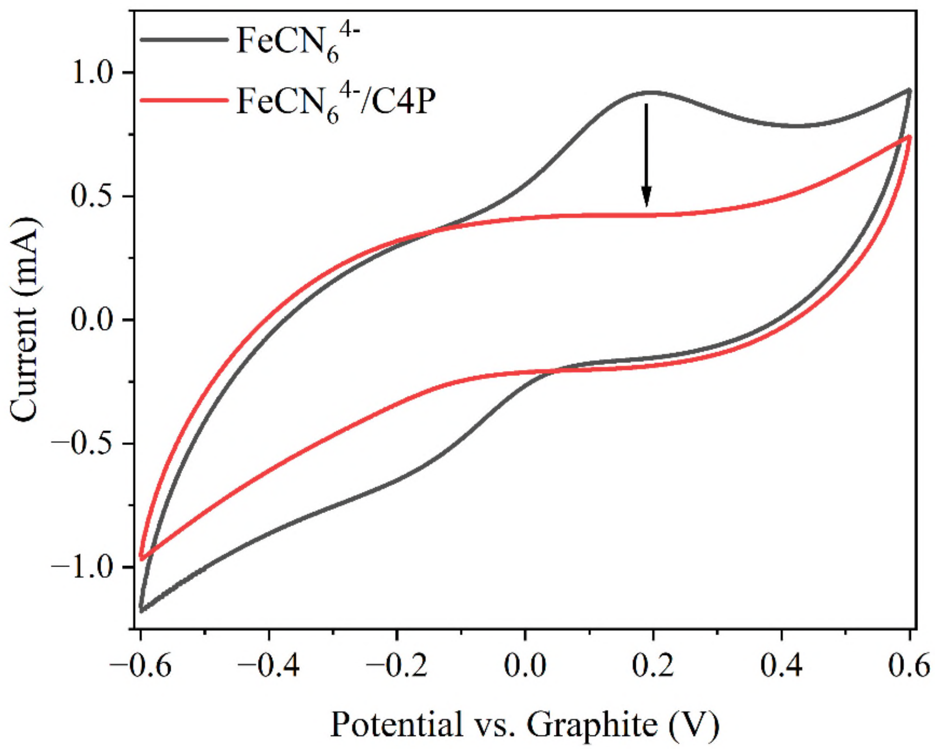

Supplementary Fig 26. Changes in CV curves of $Fe(CN)_6^{4-}$ upon addition of C4P.

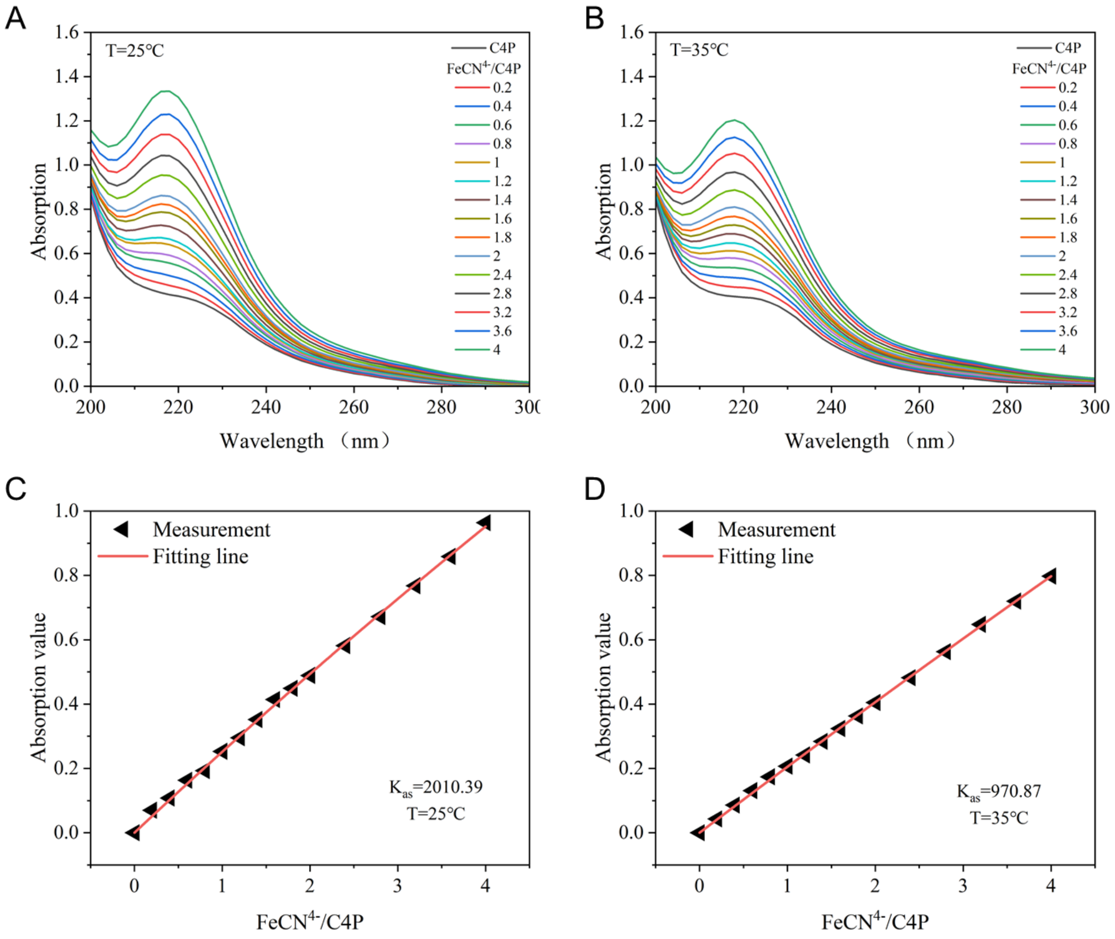

Supplementary Fig 27. Determination of the complexation constant between C4P and $Fe(CN)_6^{4-}$ at different temperatures. (A, C) UV-Vis spectra of C4P and $Fe(CN)_6^{4-}$ mixtures at varying molar ratios at two different temperatures. (B, D) Calculation of the complexation constants based on UV adsorbance fitting. The results indicate that the binding affinity between C4P and $Fe(CN)_6^{4-}$ decreases with increasing temperature, consistent with thermally induced dissociation of the host–guest complex.

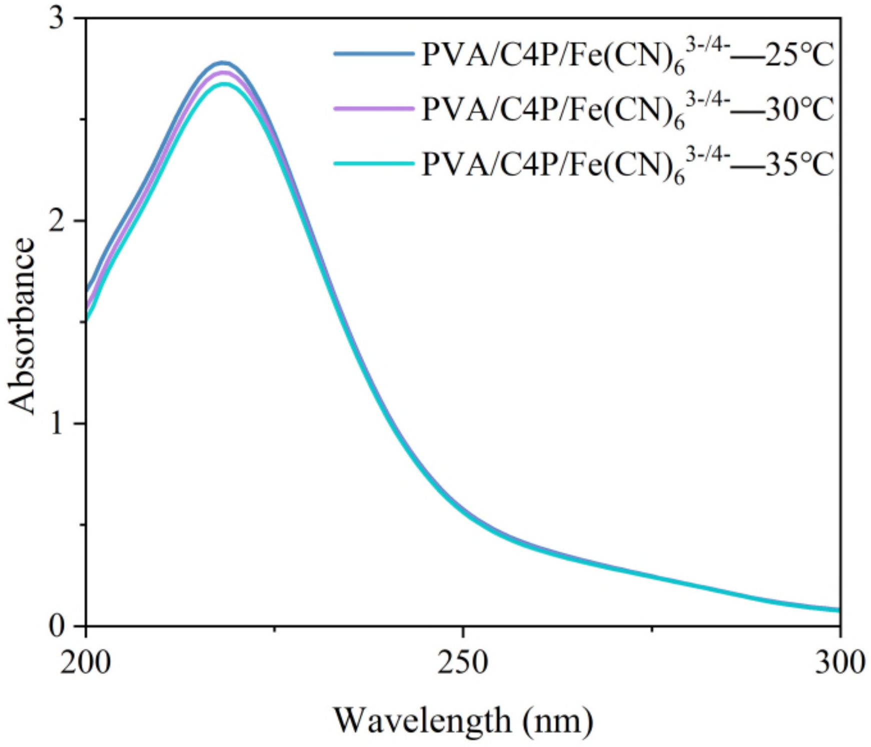

Supplementary Fig 28. UV-Vis spectra of PVA/C4P/$Fe(CN)_6^{3-/4-}$ system at different temperatures. As the temperature increases, the $Fe(CN)_6^{4-}$@C4P complex undergoes gradual decomplexation, releasing free $Fe(CN)_6^{4-}$ and thereby facilitating the continuous conversion of $Fe(CN)_6^{4-}$ to $Fe(CN)_6^{3-}$ at the hot end of the thermoelectric cell (TEC).

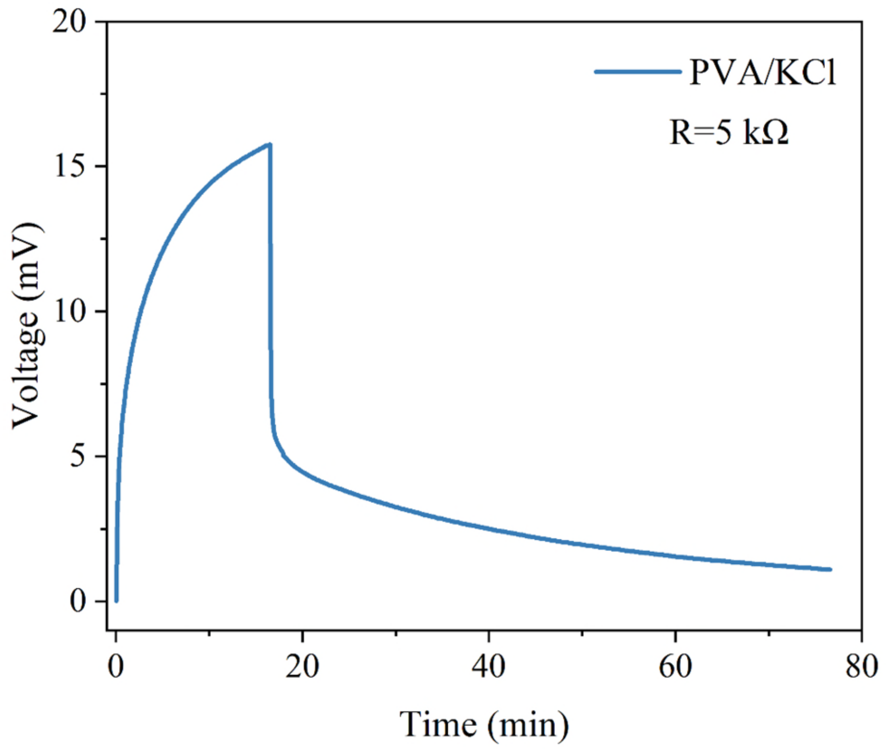

Supplementary Fig 29. Voltage variation curve of PVA/KCl at a load of 5 kΩ. The temperature difference is 10 K.

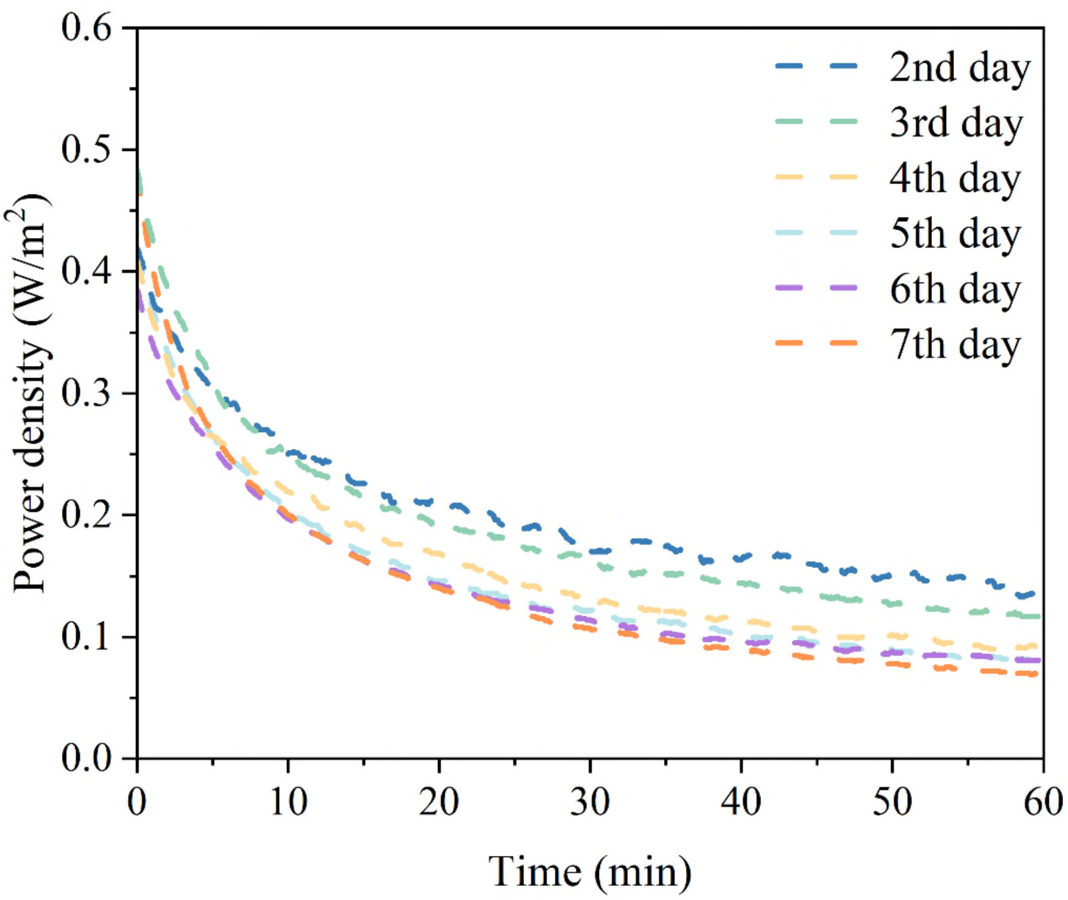

Supplementary Fig 30. Stability assessment of the PVA/C4P/KCl/$Fe(CN)_6^{3-/4-}$ thermoelectric cell, discharged continuously for 1 hour per day over a period of 7 consecutive days under a constant load of 100 Ω.

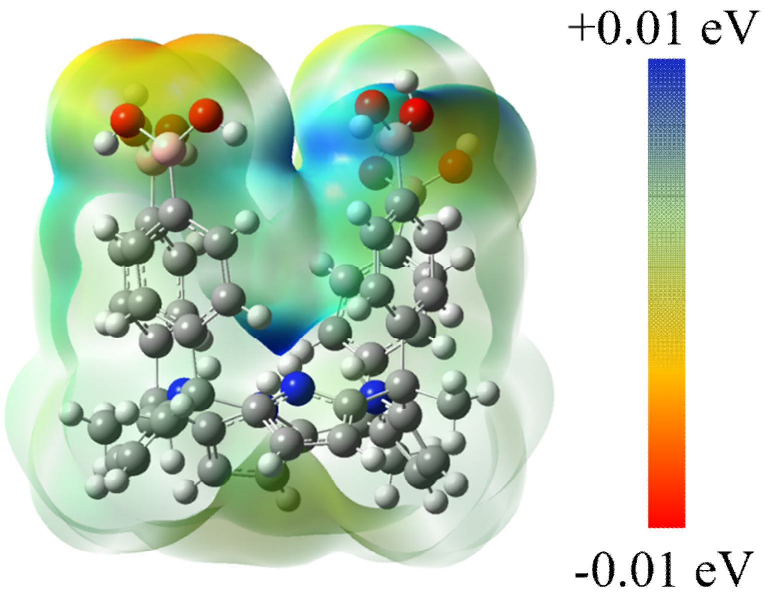

Supplementary Fig 31. Electrostatic potential (ESP) map of C4P. It indicates that the -NH functional group within the C4P cavity carries a positive charge of approximately 0.01 eV. This positive charge increases its ability to interact with negatively charged species like $Fe(CN)_6^{4-}$.

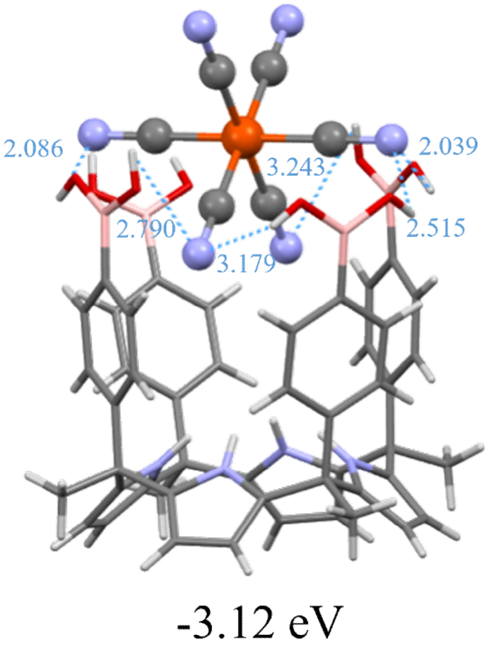

Supplementary Fig 32. Simulated host–guest interaction between C4P and $Fe(CN)_6^{4-}$, illustrating the binding mode and stabilizing hydrogen-bond interactions within the macrocyclic cavity.

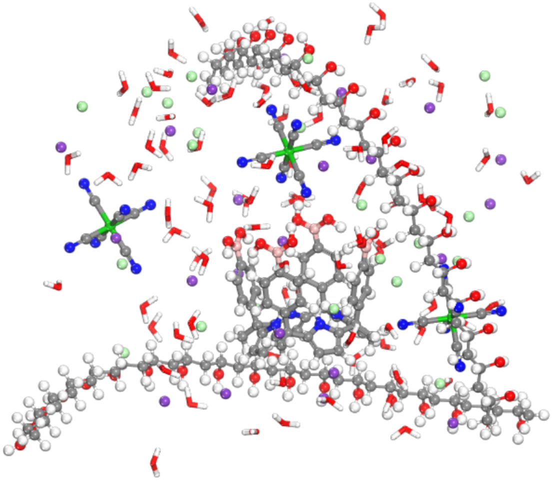

Supplementary Fig 33. Molecular dynamics simulation of the dissociation behavior of $Fe(CN)_6^{4-}$ in the PVA hydrogel system.

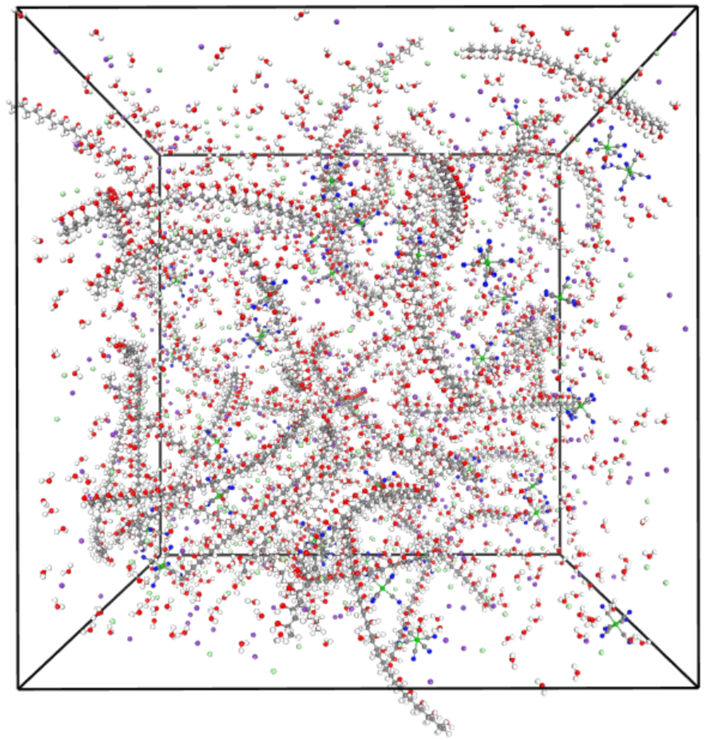

Supplementary Fig 34. Simulated ion distribution in the PVA hydrogel system before the addition of C4P.

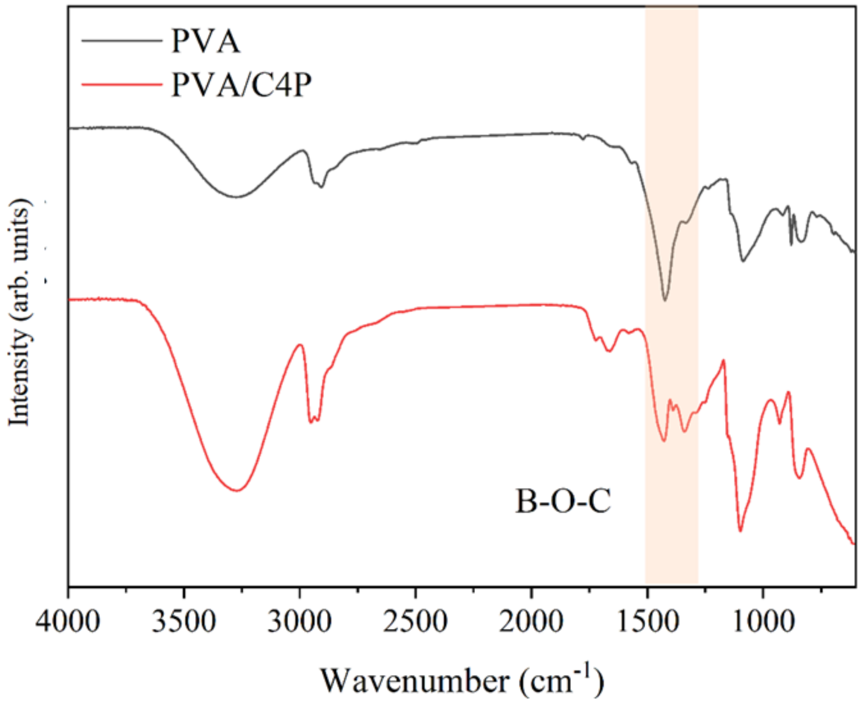

Supplementary Fig 35. FTIR spectrum of PVA/C4P, showing characteristic features of crosslinking between C4P and PVA. The formation of dynamic borate (boronic ester) bonds confirms intermolecular interactions that contribute to the structural integrity of the gel.

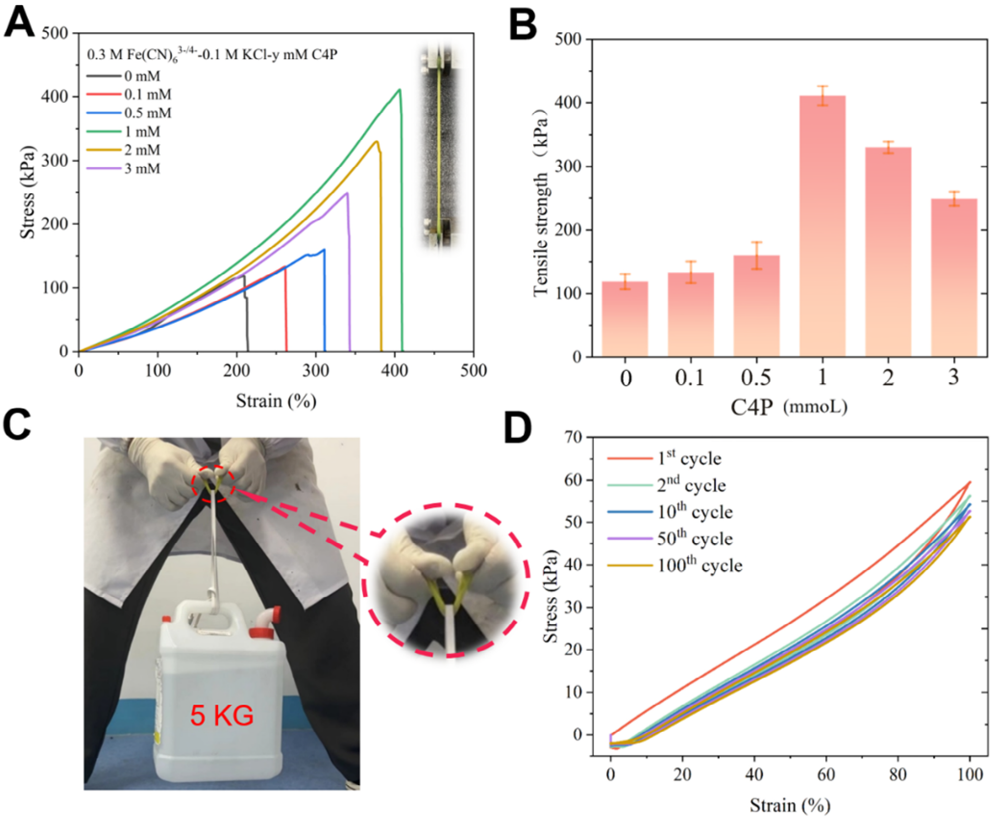

Supplementary Fig 36. Mechanical properties of gel TECs. (A) Stress-strain curves of gel TECs with various C4P concentrations. (B) Tensile stresses obtained with various C4P concentrations with tensile speed of 100 mm/min. Data are presented as mean values ± SD (n = 3). (C) Demonstration of high fracture strength of TEC with 1 mM C4P addition (hydrogel size: 2 × 5 cm; barrel weight: 5 kg). (D) Continuous tensile loaded-unloaded stress-strain curve of TEC with 1 mM C4P at 100% strain

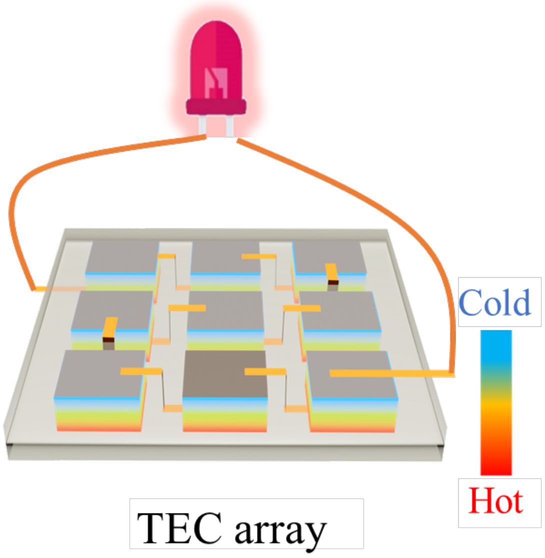

Supplementary Fig 37. Schematic diagram of an integrated thermoelectric conversion module with nine gel TEC units.

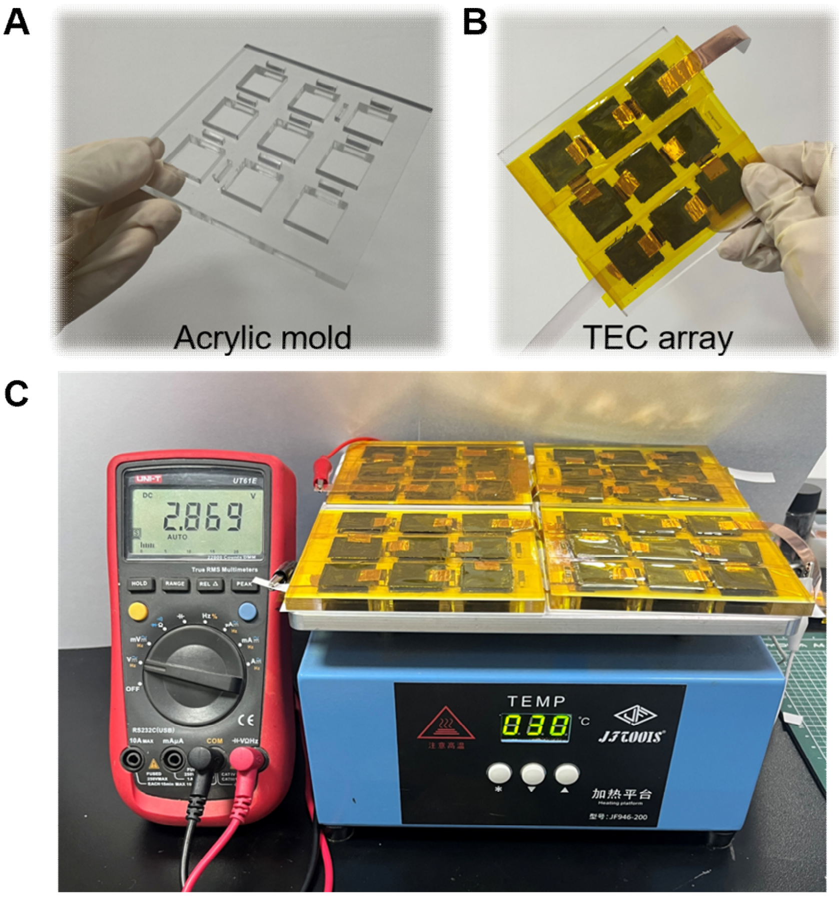

Supplementary Fig 38. TEC arrays assembly and performance test. (A) Real image of an acrylic mould. (B) Real image of a single TEC array after assembly. (B) Image of open circuit voltage at ~ 10 K temperature difference after testing 4 arrays in series. It is important to note that the temperatures displayed on the heated table instrumentation are for reference only; during the test the temperatures were measured using type K thermocouples to determine the precise temperature difference between the hot and cold ends of the TECs. In Supplementary Fig 21(C), the temperature of the hot end of the array was measured to be 30.6 °C and the temperature of the cold end was 20.7 °C.

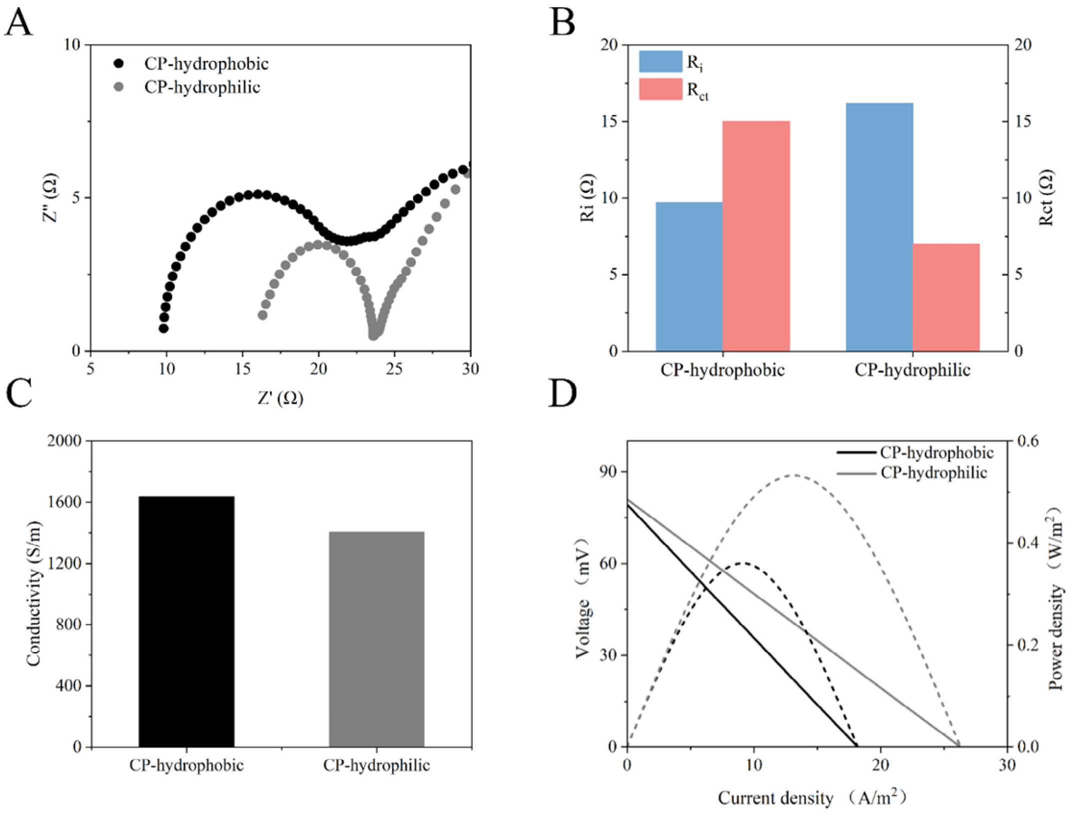

Supplementary Fig 39. Comparative electrochemical performance of untreated carbon paper (CP-hydrophobic) and hydrophilic carbon paper (CP-hydrophilic). (A) EIS spectra of the two electrodes. (B) Internal resistance (R_i) and charge-transfer resistance (R_{ct}) values extracted from EIS data. (C) Conductivity of the two electrodes, measured by four-probe conductivity measurement. (D) I-V curves of two types of carbon paper.

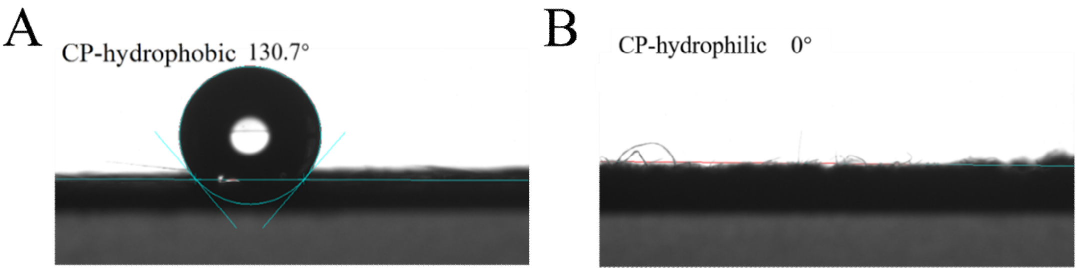

Supplementary Fig 40. Water contact angle of two types of electrodes. (A) Hydrophobic carbon paper. (B) Hydrophilic carbon paper.

Thermoelectric performance tests with Au@Cu electrodes

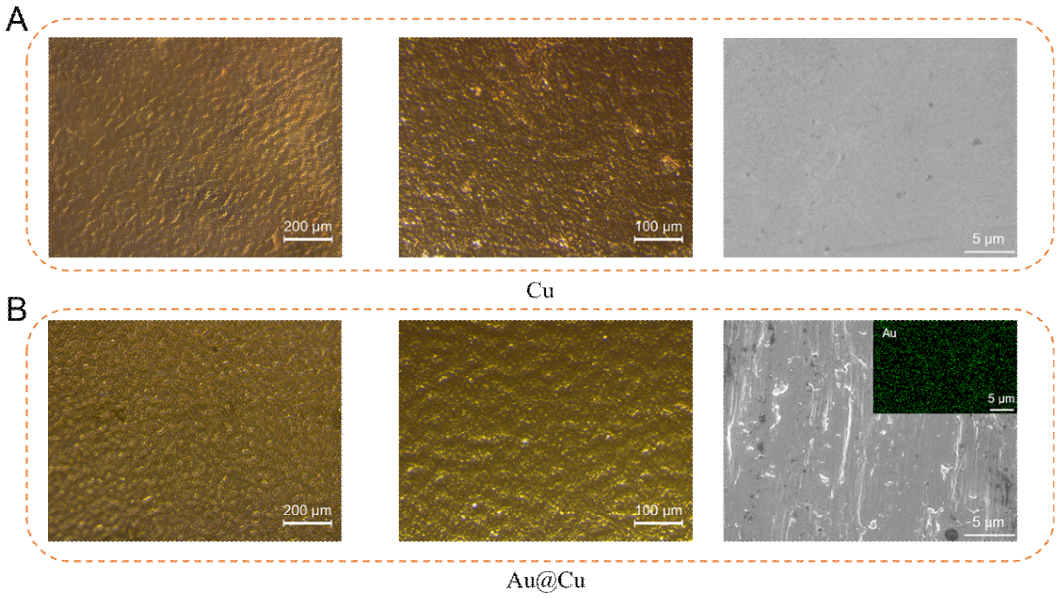

Supplementary Fig 41. Microscope and SEM images of the electrodes. (A) Microscope and SEM images of the copper foil. The surface of the copper foil is smooth and shows an orange-brown color under the optical microscope. (B) Microscope and SEM images of the copper foil after gold plating. The gold plating is as smooth as the original copper foil, with a relatively more yellow hue under the optical microscope. Further SEM observation shows that the gold plating layer adheres well to the copper foil substrate, with Au atoms densely distributed on the copper foil, as confirmed by EDX.

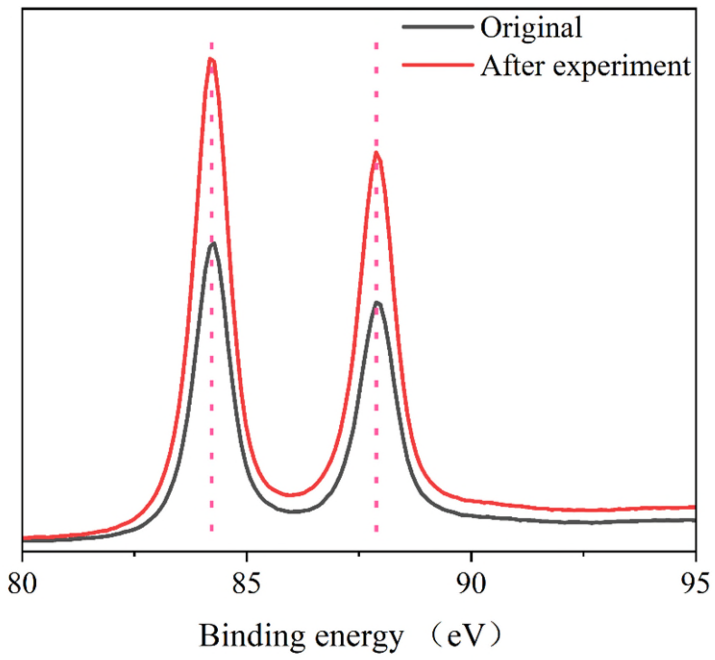

Supplementary Fig 42. XPS energy spectra of electrode gold elements before and after the experiment.

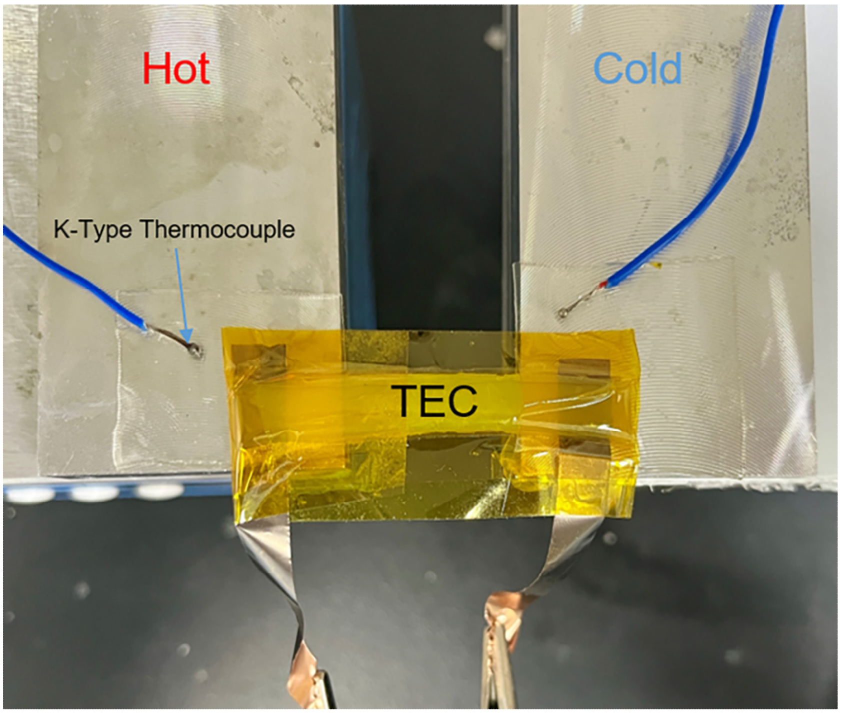

Supplementary Fig 43. Real picture of the cell thermoelectric performance test.

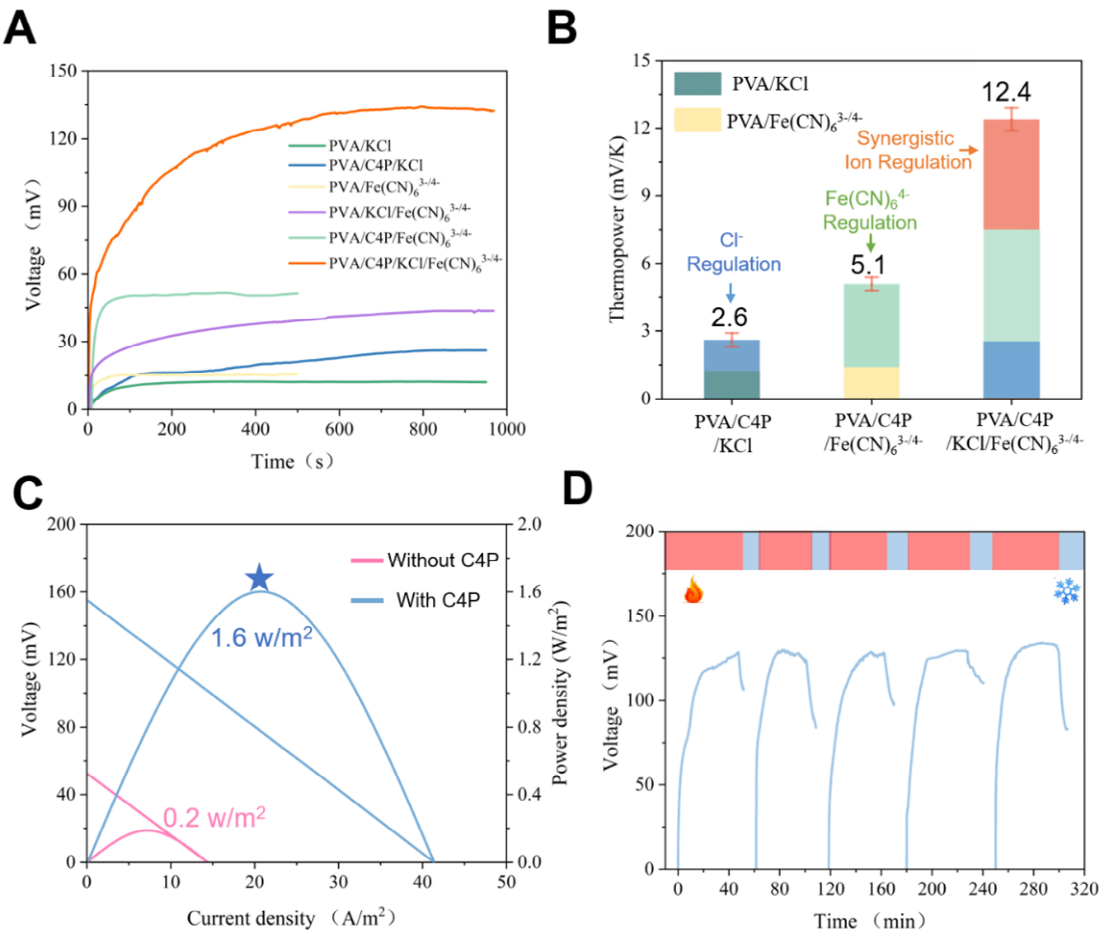

Supplementary Fig 44. Properties of the gel formed beneath the copper electrode**.** (A) Voltage-time profiles for gel TECs, measuring with gels of dimensions 1 × 5 × 0.5 cm, positioned 2 cm apart between the hot and cold ends under a controlled temperature differential (ΔT) of 10 K. (B) Comparison of the thermopower across various as-fabricated TECs. Data are presented as mean values ± SD (n = 3). (C) Current-voltage curves and corresponding power densities for PVA/KCl/$Fe(CN)_6^{3-/4-}$ and PVA/C4P/KCl/$Fe(CN)_6^{3-4-}$. (D) Open-circuit voltage response versus time curves of PVA/C4P/KCl/$Fe(CN)_6^{3-/4-}$ TEC across five thermal cycles

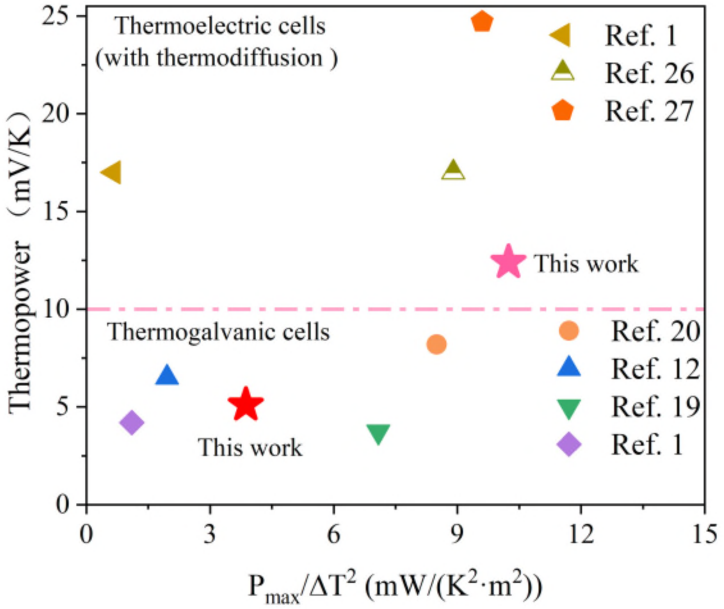

Supplementary Fig 45. Comparison of the present work with other previously reported work in terms of thermopower and normalized power density (Supplementary Table 2).

Correlation analyses and complementary experiments were conducted to clarify the influence of electrode materials on the thermoelectric properties of gels (Supplementary Figs 44–47).

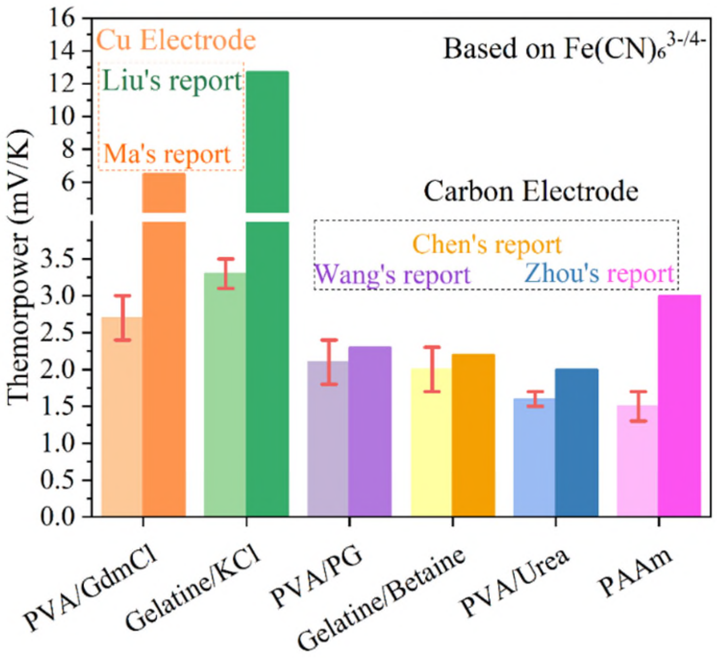

Supplementary Fig 46. Comparison of thermopower values for various $Fe(CN)_6^{3-/4-}$-based enhancement strategies tested with carbon or copper electrodes, alongside values reported in the literature. While copper electrodes consistently yield higher thermopower, the results obtained with carbon electrodes align closely with our experimental findings. Notably, reported systems incorporating urea or PAAm involved very high concentrations (>10 mol/L) in aqueous solutions, conditions that are incompatible with gel-phase systems, thereby further amplifying the discrepancy between their reported performance and our gel-phase measurements. Ma: Adv. Mater. 2023, 35, 2300696; Liu: Science 2020, 368, 1091-1098; Wang: Adv. Funct. Mater. 2023, 33, 2306509; Chen: Angew. Chem. Int. Ed. 2024, 63, e202405357; Zhou: Nat. Commun. 2018, 9, 5146-5153. Data are presented as mean values ± SD (n = 3).

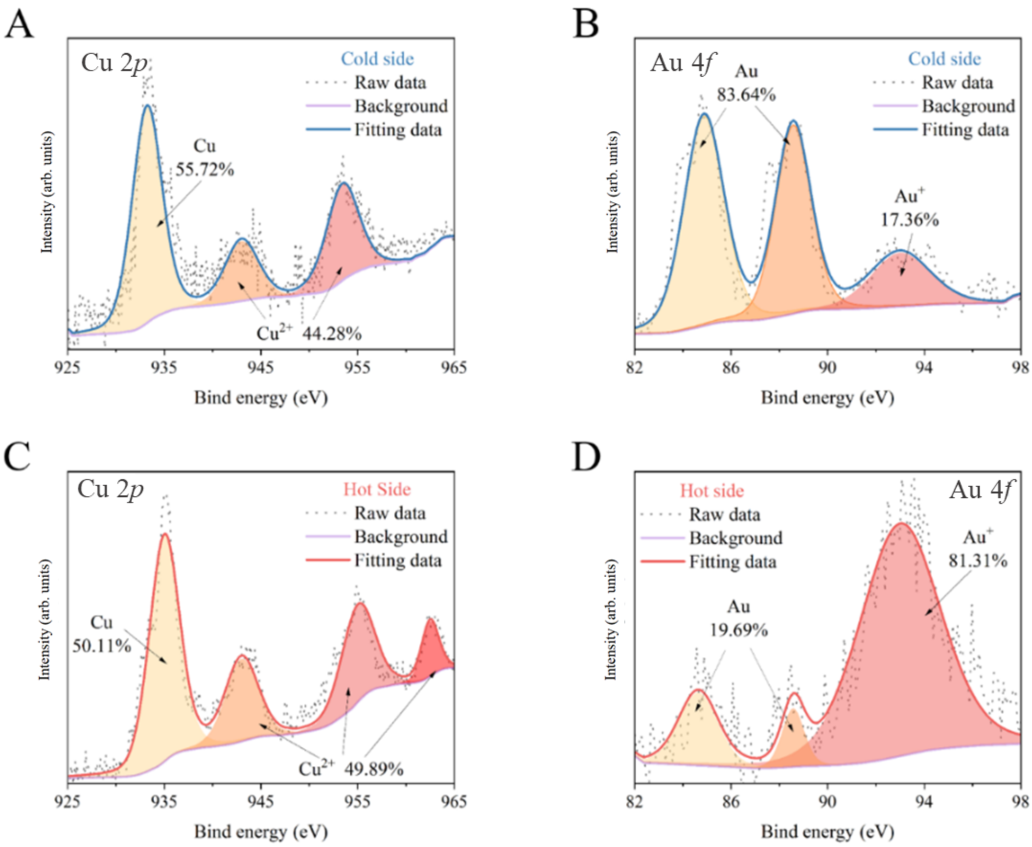

Supplementary Fig 47. XPS spectra of Cu and Au electrodes. (A,B) XPS spectra of (A) Cu and (B) Au at the cold end electrodes. (C,D) XPS spectra of (C) Cu and (D) Au at the hot end electrodes. XPS spectra show that both Au and Cu signals shift after operation, with evident oxidation signatures, more severe at the hot end than the cold end. This spatial gradient suggests that the $Fe(CN)_6^{3-/4-}$ redox couple initiates surface corrosion at the Au interface, eventually exposing and reacting with the underlying Cu layer.

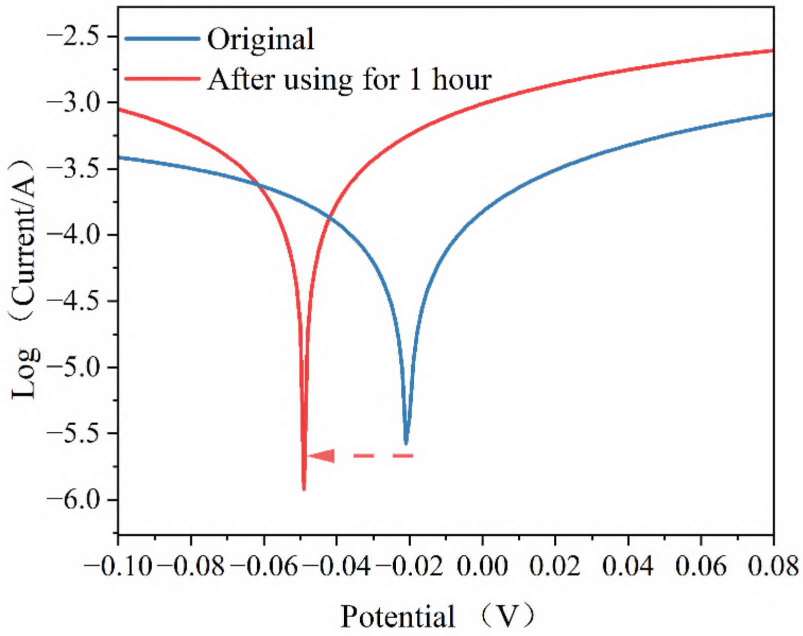

Supplementary Fig 48. Tafel curves of Cu@Au electrodes in $Fe(CN)_6^{3-/4-}$, with Pt as the reference electrode. The gradual shift in corrosion potential with immersion time confirms that reactions are actively occurring at the Cu@Au surface.

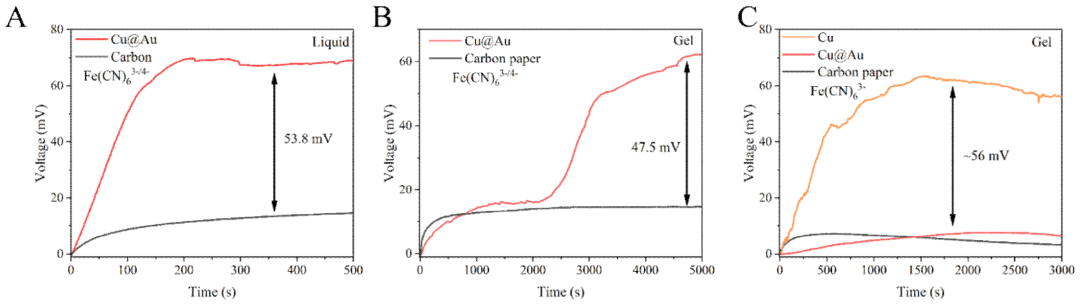

Supplementary Fig 49. Voltage curves tested under different conditions. (A) Aqueous solution of $Fe(CN)_6^{3-/4-}$, (B) $Fe(CN)_6^{3-/4-}$ hydrogel, (C) $Fe(CN)_6^{3-}$ hydrogel. The temperature difference was 10 K in all cases, and the ion concentration was 0.3 M. The hydrogel's high water-retention capacity significantly suppresses the local activity of $Fe(CN)_6^{3-/4-}$ ions at the electrode–gel interface. This results in a lower effective ion concentration near the electrode surface, thereby attenuating both redox kinetics and corrosion reactions; Prior to the complete degradation of the Au overlayer, the Cu@Au electrode behaves effectively as an inert conductor. Under these conditions, the measured thermopower predominantly reflects the intrinsic Seebeck coefficient of the redox couple (~1.4 mV K^{-1}), free from interference by parasitic redox currents. However, in systems involving significant thermodiffusion, the slower approach to voltage equilibrium affords a longer window for corrosion to occur. As a result, the corrosion process, particularly Cu oxidation, introduces an additional bias (~50 mV), which becomes superimposed on the true thermovoltage, leading to an overestimation of performance metrics.

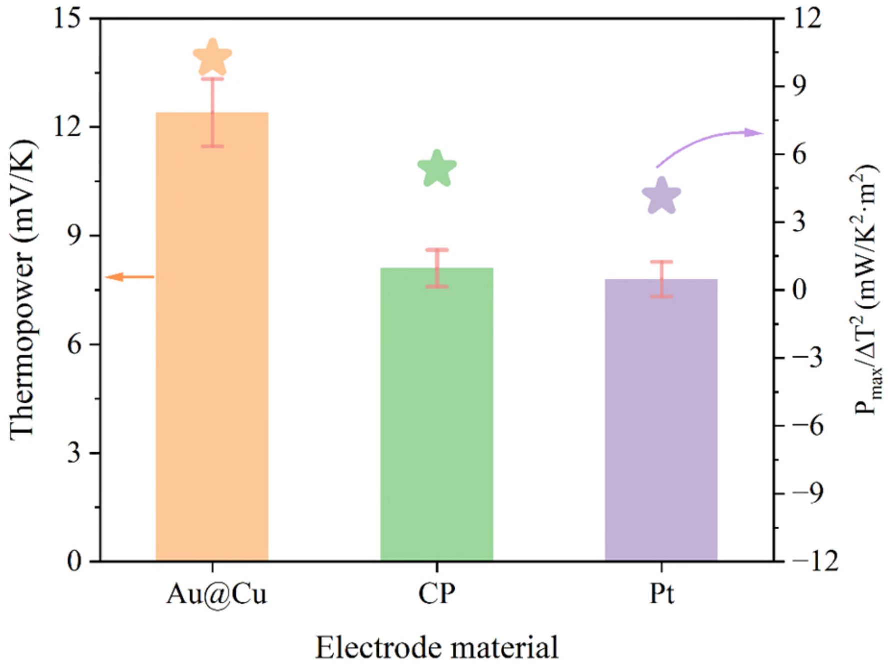

Supplementary Fig 50. Comparison of thermopower and normalized power for cells employing different electrodes. Using Au@Cu electrodes results in additional apparent thermoelectric output due to electrode corrosion, contributing an extra 4.3 mV K^{-1} (approximately 34.7 % of the total thermopower). In contrast, cells assembled with carbon or platinum electrodes exhibit nearly identical performance. Data are presented as mean values ± SD (n = 3).

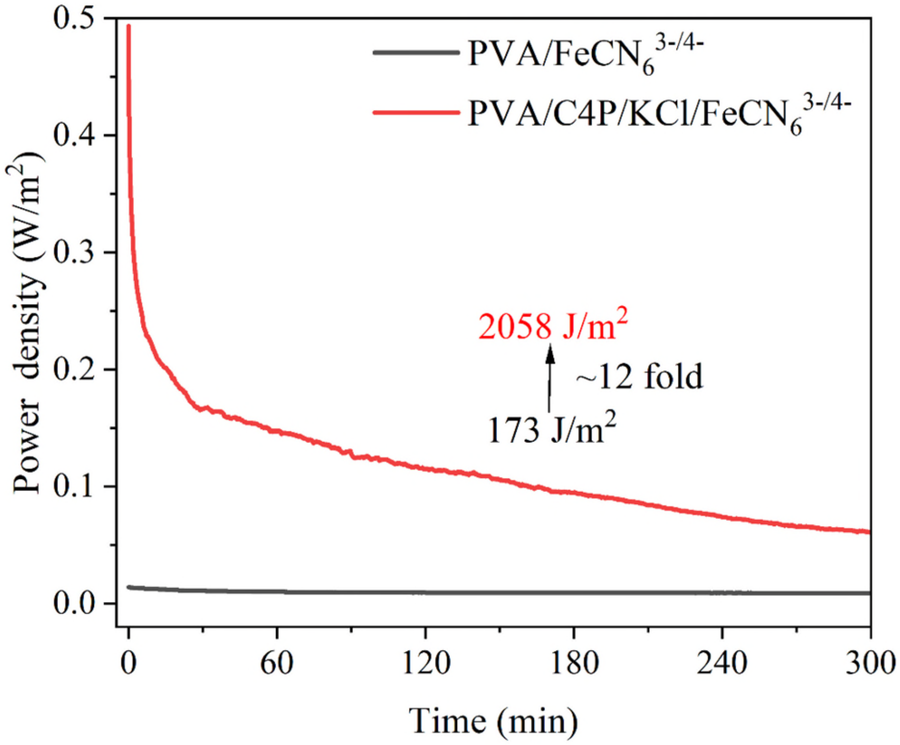

Supplementary Fig 51. Comparison of energy output variation between two types of gel-based thermogalvanic cells during discharge across a 100 Ω load under a 10 K temperature difference for 5 h. The PVA/C4P/KCl/$Fe(CN)_6^{3-/4-}$ thermocell maintains markedly higher output power than the PVA/$Fe(CN)_6^{3-/4-}$ system throughout the discharge period. Over 5 h, its total energy output is approximately twelve times greater, demonstrating the enhanced stability and sustained energy conversion efficiency achieved through dual-anion regulation.

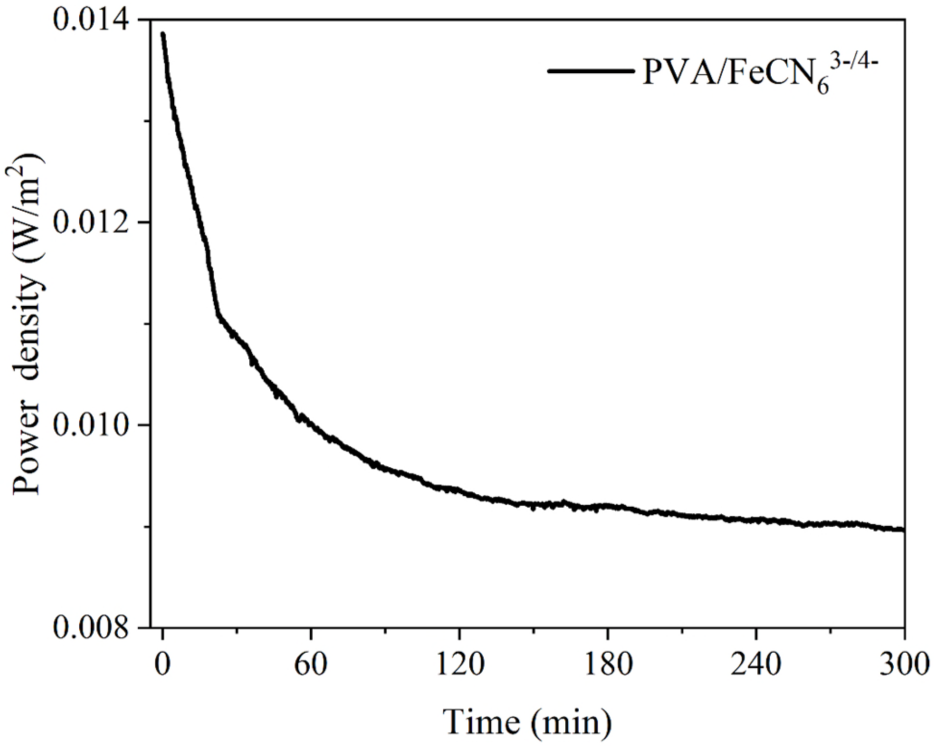

Supplementary Fig 52. Enlarged view of the PVA/Fe(CN)$_6^{3-/4-}$ cell from Supplementary Fig 48. Notably, even the purely thermogalvanic system exhibits inevitable power decay during prolonged discharge.

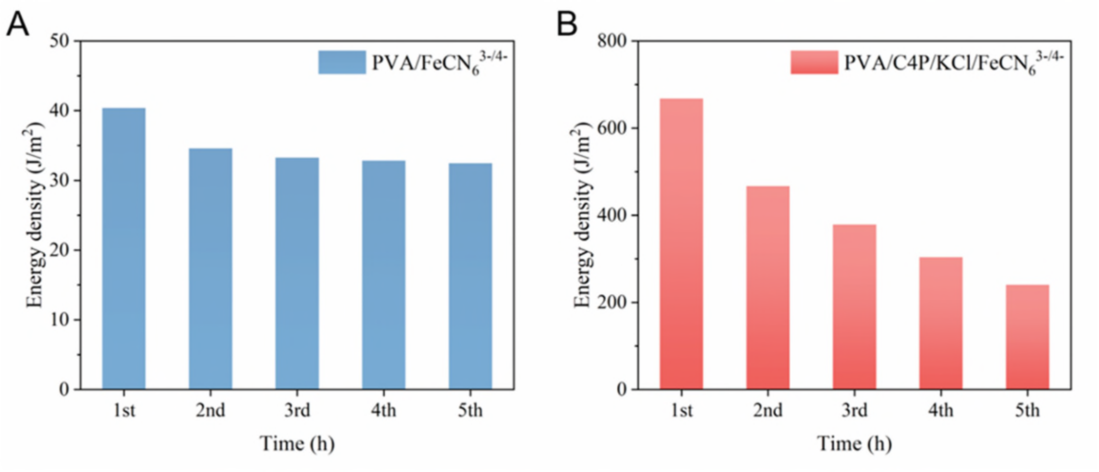

Supplementary Fig 53. Comparison of the energy density released per hour for two gel-based thermalcells, calculated from the discharge profiles in Supplementary Fig 49. (A) PVA/$Fe(CN)_6^{3-/4-}$ cell; (B) PVA/C4P/KCl/$Fe(CN)_6^{3-/4-}$ cell.

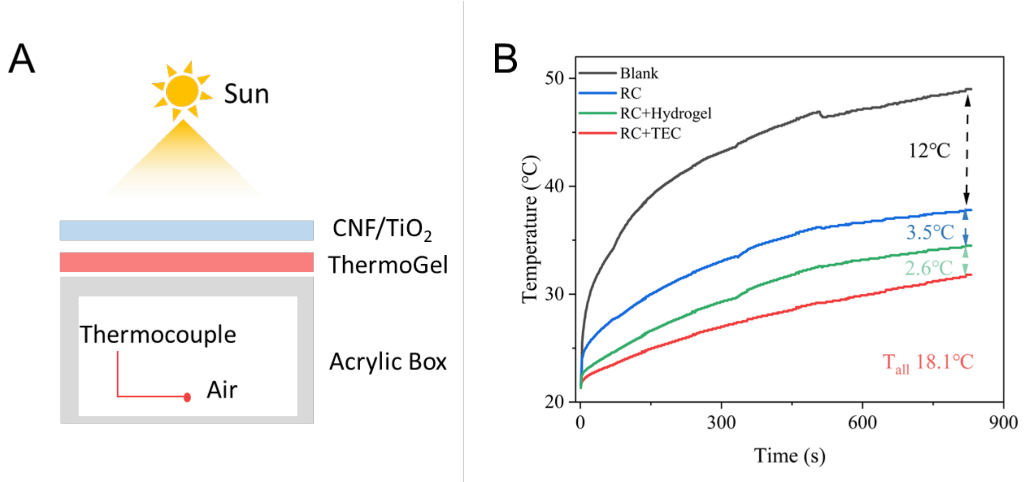

Supplementary Fig 54. TEC for building thermal management. (A) Schematic illustration of the scaled model integrating the TEC with a radiative cooling film for active–passive hybrid heat dissipation. (B) Comparison of interior temperature evolution under different configurations, demonstrating enhanced cooling performance of the RC/TEC system. A cubic acrylic chamber (5 cm × 5 cm × 2 cm) was used to simulate a building interior under solar illumination (1 sun, 1000 W/m^2). The internal air temperature was monitored by a thermocouple placed at the center of the chamber. Four configurations were tested: (i) bare chamber (blank), (ii) chamber covered with a radiative cooling (RC) film composed of cellulose nanofiber (CNF) and TiO_2, (iii) RC film laminated with pure PVA hydrogel, and (iv) RC film integrated with the TEC. Under steady-state conditions, the RC/TEC system achieved the lowest interior temperature. Compared to the RC/PVA hydrogel reference, the TEC integration provided an additional cooling of 2.6 °C, demonstrating that thermal-to-electrical conversion actively enhances heat removal. Relative to the bare chamber, the total temperature reduction reached 18.1 °C

Supplementary Tables

Supplementary Table 1. Comprehensive TEC performance comparison

Matrix	S_e (mV/K)	$P_{max}/(\Delta T)^2$ ($mW/K^2 \cdot m^2$)	σ (S/m)	1/κ (m·K/W)	1/cost	Ref.
PVA/C4P/KCl /$FeCN^{3-/4-}$	8.1	5.33	3.25	2.78	8.33	This work
Gelatin/Betaine /$FeCN^{3-/4-}$	2.2	0.48	3.5	N/A	4.27	(*4*)
Cellulose/GdmCl /LiCl/$FeCN^{3-/4-}$	3.42	2.8	2.52	N/A	0.54	(5)
Cellulose/PG /$FeCN^{3-/4-}$	2.3	0.35	5.27	1.21	6.67	(3)
PVA/Agar /$FeCN^{3-/4-}$	1.5	0.4	0.66	N/A	5	(*6*)

⁎ We calculate the cost in CNY Yuan. In the cost analysis, only the materials directly responsible for performance enhancement, such as C4P in this work, were considered. The loading of each additive was taken from the optimal composition reported in the corresponding literature and normalized to the amount required to prepare 10 g of gel. All costs were calculated based on the listed prices from the Energy Chemical Platform.

Supplementary Table 2. Comparison of the thermopower (S_e), $P_{max}/(\Delta T)^2$ and σ of this work with those reported for TECs in the literature

Matrix	Electrode	Redox couple	S_e (mV/K)	$P_{max}/(\Delta T)^2$ ($mW/K^2 \cdot m^2$)	σ (S/m)	Ref.
PVA/C4P/KCl	Carbon	$FeCN^{3-/4-}$	8.1	5.33	3.25	This work
Ov-WO_3/Polyacrylic acid/Sv-ZIS	Au@Cu	$FeCN^{3-/4-}$	8.2	8.5	4.7	(*7*)
PVA/GdmCl	Cu	$FeCN^{3-/4-}$	6.5	1.96	6	(*8*)
H_2O	Carbon	$FeCN^{3-/4-}$	2.9	0.64	15	(*9*)
Polyacrylamide	Cu	$FeCN^{3-/4-}$	1.5	0.61	12	(*10*)
H_2O	Carbon	$FeCN^{3-/4-}$	1.43	0.01	N/A	(*12*)
Polyacrylamide	Cu	$Fe^{2+/3+}$	2.02	0.012	1.47	(*11*)
PVA/Agar	Carbon	$FeCN^{3-/4-}$	1.5	0.4	0.66	(*6*)
H_2O	Carbon	$FeCN^{3-/4-}$	1.42	1.9	N/A	(*13*)
H_2O	Carbon	$FeCN^{3-/4-}$	1.43	0.6	N/A	(*14*)
H_2O	Carbon	$FeCN^{3-/4-}$	1.3	0.36	0.51	(*15*)
Polyacrylamide	Carbon	$Fe^{2+/3+}$	1.21	0.04	1.1	(*16*)
Polyacrylamide/GdmCl	Pt	$FeCN^{3-/4-}$	4.4	1.78	10.2	(*17*)
Polyacrylic acid/cellulose	Carbon	$FeCN^{3-/4-}$	1.3	0.033	N/A	(*18*)
PVA/gelation	Carbon	$FeCN^{3-/4-}$	2.02	0.1	1	(*19*)
PVA	Cu	$FeCN^{3-/4-}$	1.5	0.22	6.3	(*20*)
H_2O	Carbon	$FeCN^{3-/4-}$	1.4	0.5	N/A	(*21*)
Cellulose	Pt	$FeCN^{3-/4-}$	1.4	0.144	N/A	(*22*)
H_2O	Carbon	$FeCN^{3-/4-}$	4.2	1.1	24	(*23*)
H_2O	Carbon	$FeCN^{3-/4-}$	3.73	7.08	36	(*24*)
H_2O	Carbon	$FeCN^{3-/4-}$	1.45	0.12	N/A	(*25*)
Gelatin/KCl	Au@Cu	$FeCN^{3-/4-}$	17	1.8	N/A	(*1*)
Gelatin/KCl	Au@Cu	$FeCN^{3-/4-}$	17	8.9	N/A	(*26*)
Gelatin/KCl	Au@Cu	$FeCN^{3-/4-}$	24.7	9.6	N/A	(*27*)
Poly (N,N-dimethylacrylamide)/ ([EMIM][DCA])	Al	$FeCN^{3-/4-}$	32.4	25.84	3.7	(*28*)

Supplementary References

1 Han, C. G. *et al.* Giant thermopower of ionic gelatin near room temperature. *Science* **368**, 1091-1098 (2020).

2 Agar, J. N., Mou, C. Y., Lin, J. L. Single-ion heat of transport in electrolyte solutions: a hydrodynamic theory. *J. Phys. Chem.* **93**, 2079-2082 (1989).

3 Li, J. *et al.* High Performance Bacterial Cellulose Organogel-Based Thermoelectrochemical Cells by Organic Solvent-Driven Crystallization for Body Heat Harvest and Self-Powered Wearable Strain Sensors. *Adv. Funct. Mater.* **33**, 2306509 (2023).

4 Pang, B. Liu, H. Zhang, K. Recent progress on Pickering emulsions stabilized by polysaccharides-based micro/nanoparticles. *Adv. Colloid Interface Sci.* **296**, 102522 (2021).

5 Gui, J. X. *et al.* Development of Ternary Hydrogel Electrolytes for Superior Gel Thermocells: Exceptional Anti-Drying, Anti-Freezing, and Mechanical Robustness. *Adv.Mater.* **37**, 2420214 (2025).

6 Ma, X. L. et al. Machine learning assisted self‐powered identity recognition based on thermogalvanic hydrogel for intelligent security. *Small* **20**, 2402700 (2024).

7 Wang, Y. J. *et al.* In situ photocatalytically enhanced thermogalvanic cells for electricity and hydrogen production. *Science* **381**, 291-296 (2023).

8 Liu, L. et al. Strong tough thermogalvanic hydrogel thermocell with extraordinarily high thermoelectric performance. *Adv. Mater.* **35**, 2300696(2023).

9 Kim, T. et al. High thermopower of ferri/ferrocyanide redox couple in organic-water solutions. *Nano Energy* **31**, 160-167 (2017).

10 Lei, Z., Gao, W. & Wu, P. Double-network thermocells with extraordinary toughness and boosted power density for continuous heat harvesting. *Joule* **5**, 2211-2222 (2021).

11 Gao, W., Lei, Z., Zhang, C., Liu, X. & Chen, Y. Stretchable and freeze‐tolerant organohydrogel thermocells with enhanced thermoelectric performance continually working at subzero temperatures. *Adv. Funct. Mater.* **31**, 2104071(2021).

12 Kang, T. J. et al. Electrical power from nanotube and graphene electrochemical thermal energy harvesters. *Adv. Funct. Mater.* **22**, 477-489 (2011).

13 Romano, M. S. *et al.* Carbon nanotube – reduced graphene oxide composites for thermal energy harvesting applications. *Adv. Mater.* **25**, 6602-6606 (2013).

14 Zhang, L. *et al.* High power density electrochemical thermocells for inexpensively harvesting low‐grade thermal energy. *Adv. Mater.* **29**, 1605652 (2017).

15 Li, G. *et al.* High‐efficiency cryo‐thermocells assembled with anisotropic holey graphene aerogel electrodes and a eutectic redox electrolyte. *Adv. Mater.* **31**, e1901403 (2019).

16 Yang, P. H. *et al.* Wearable thermocells based on gel electrolytes for the utilization of body heat. *Angew. Chem. Int. Ed.* **55**, 12050-12053 (2016).

17 Zhang, D. *et al.* Stretchable thermogalvanic hydrogel thermocell with record-high specific output power density enabled by ion-induced crystallization. *Energy Environ. Sci.* **15**, 2974-2982 (2022).

18 Shen, J. *et al.* Boosting solar-thermal-electric conversion of thermoelectrochemical cells by construction of a carboxymethylcellulose-interpenetrated polyacrylamide network. *J. Mater. Chem. A* **10**, 7785-7791 (2022).

19 Bai, C. *et al.* Transparent stretchable thermogalvanic PVA/gelation hydrogel electrolyte for harnessing solar energy enabled by a binary solvent strategy. *Nano Energy* **100**, 107449 (2022).

20 Gao, W., Lei, Z., Chen, W. & Chen, Y. Hierarchically anisotropic networks to decouple mechanical and ionic properties for high-performance quasi-solid thermocells. *ACS Nano* **16**, 8347-8357 (2022).

21 Hu, R. *et al.* Harvesting waste thermal energy using a carbon-nanotube-based thermo-electrochemical cell. *Nano Lett.* **10**, 838-846 (2010).

22 Jin, L. Y. *et al.* Redox-active quasi-solid-state electrolytes for thermal energy harvesting. *ACS Energy Lett.* **1**, 654-658 (2016).

23 Duan, J. J. *et al.* Aqueous thermogalvanic cells with a high seebeck coefficient for low-grade heat harvest. *Nat. Commun* **9**, 5146-5153 (2018).

24 Yu, B. Y. *et al.* Thermosensitive crystallization-boosted liquid thermocells for low-grade heat harvesting. *Science* **370**, 342-346 (2020).

25 Yang, Y. *et al.* Charging-free electrochemical system for harvesting low-grade thermal energy. *Proc. Natl. Acad. Sci. U.S.A.* **111**, 17011-17016 (2014).

26 Li, Y. C. *et al.* 3D hierarchical electrodes boosting ultrahigh power output for gelatin-KCl- $FeCN^{4-/3-}$ ionic thermoelectric cells. *Adv. Energy Mater.* **12**, 2103666 (2022).

27 Li, Y. C. *et al.* Realizing record-high output power in flexible gelatin/GTA-KCl-$FeCN^{4-/3-}$ ionic thermoelectric cells enabled by extending the working temperature range. *Energy Environ. Sci.* **15**, 5379-5390 (2022).

28 Yang, M. C. *et al.* Chaotropic effect-boosted thermogalvanic ionogel thermocells for all-weather power generation. *Adv. Mater.***36**, 2312249(2024).
