# Supplementary material for: Synergistic dual anion regulation unlocks giant thermopower and power density in hydrogel
Source: Nat Commun. 2026 Mar 30;17:4592. doi: 10.1038/s41467-026-71285-3 (PMC13194681; doi:10.1038/s41467-026-71285-3)
Supplement: Supplementary file 2 — Description of Additional Supplementary Files [file 41467_2026_71285_MOESM2_ESM.pdf]

Description of Additional Supplementary Files

File name: Supplementary Movie 1

Description: The molecular dynamic process of complexation between C4P and $Fe(CN)_6^{4-}$.

File name: Supplementary Movie 2

Description: The molecular dynamic process of dissociation between C4P and $Fe(CN)_6^{4-}$.

File name: Supplementary Movie 3

Description: Demonstration of the point-touch human-computer interaction device.
